# Irreversible electroporation augments checkpoint immunotherapy in prostate cancer and promotes tumor antigen-specific tissue-resident memory CD8+ T cells

**DOI:** 10.1038/s41467-021-24132-6

**Published:** 2021-06-23

**Authors:** Brandon J. Burbach, Stephen D. O’Flanagan, Qi Shao, Katharine M. Young, Joseph R. Slaughter, Meagan R. Rollins, Tami Jo L. Street, Victoria E. Granger, Lalit. K. Beura, Samira M. Azarin, Satish Ramadhyani, Bruce R. Forsyth, John C. Bischof, Yoji Shimizu

**Affiliations:** 1 Department of Laboratory Medicine and Pathology, University of Minnesota, Minneapolis, MN USA; 2 Center for Immunology, University of Minnesota, Minneapolis, USA; 3 Masonic Cancer Center, University of Minnesota, Minneapolis, USA; 4 Institute for Engineering in Medicine, University of Minnesota, Minneapolis, USA; 5 Department of Microbiology and Immunology, University of Minnesota, Minneapolis, USA; 6 Department of Mechanical Engineering, University of Minnesota, Minneapolis, USA; 7 Boston Scientific Corporation, Maple Grove, MN USA; 8 Department of Chemical Engineering and Materials Science, University of Minnesota, Minneapolis, USA; 9 BTG plc, Arden Hills, MN USA; 10 Department of Biomedical Engineering, University of Minnesota, Minneapolis, USA; 11 Present Address: Department of Molecular Microbiology and Immunology, Brown University, Providence, RI USA

**Keywords:** Tumour immunology, Immunotherapy, Biomedical engineering

## Abstract

Memory CD8+ T cells populate non-lymphoid tissues (NLTs) following pathogen infection, but little is known about the establishment of endogenous tumor-specific tissue-resident memory T cells (T_RM_) during cancer immunotherapy. Using a transplantable mouse model of prostate carcinoma, here we report that tumor challenge leads to expansion of naïve neoantigen-specific CD8+ T cells and formation of a small population of non-recirculating T_RM_ in several NLTs. Primary tumor destruction by irreversible electroporation (IRE), followed by anti-CTLA-4 immune checkpoint inhibitor (ICI), promotes robust expansion of tumor-specific CD8+ T cells in blood, tumor, and NLTs. Parabiosis studies confirm that T_RM_ establishment following dual therapy is associated with tumor remission in a subset of cases and protection from subsequent tumor challenge. Addition of anti-PD-1 following dual IRE + anti-CTLA-4 treatment blocks tumor growth in non-responsive cases. This work indicates that focal tumor destruction using IRE combined with ICI is a potent in situ tumor vaccination strategy that generates protective tumor-specific T_RM_.

## Abstract (web summary)

Irreversible electroporation (IRE), a soft-tissue ablation technique used for tumour ablation, has been suggested to promote systemic immune responses. Here the authors show that IRE, followed by anti-CTLA-4 blockade, elicits the expansion of tumor antigen-specific CD8+ T cells and is associated with tissue residency and improved anti-tumor immune response in a preclinical model of prostate cancer.

## Introduction

CD8+ T cells play a critical role in host immune responses to intracellular pathogens and malignancies. Upon recognition of cognate antigen presented by dendritic cells in regional lymph nodes, naive CD8+ T cells respond to infected or malignant cells by expanding in number and differentiating into effector and memory subsets that redistribute throughout the body to recognize and eliminate target cells. Clinical challenges in cancer immunotherapy include increasing the frequency of endogenous tumor antigen-specific T cells, and reversing functional exhaustion of T cells within tumors^1,2^. Therapeutic blockade of the inhibitory proteins cytotoxic T-lymphocyte associated-protein 4 (CTLA-4) and programmed cell death protein-1 (PD-1) on exhausted CD8+ T cells using immune checkpoint inhibitors (ICI) is associated with tumor remission, particularly in subsets of patients with prominent CD8+ T cell infiltration of tumors^3,4^. Despite these advances many patients are not responsive to ICI, in part due to impaired expansion and redistribution of endogenous tumor antigen-specific CD8+ T cells into tumors and non-lymphoid tissue (NLT) body sites where disseminated tumor cells may metastasize^4^.

Two subsets of recirculating memory CD8+ T cells known as central memory (T_CM_) and effector memory (T_EM_) can be sampled in the blood but a third, non-recirculating subset known as tissue-resident memory (T_RM_) is parked in NLTs, and is of increasing interest for its role in both pathogen immunity and cancer immunotherapy^5–8^. T_CM_ recirculate between blood and secondary lymphoid organs (SLO) and express CD62L and CCR7, while T_EM_ traffic between blood and NLTs and express low levels of CD62L and CCR7^9,10^. In contrast to T_CM_ and T_EM_, the T_RM_ subset of memory CD8+ T cells are non-recirculating, frequently associated with exclusion from the vasculature, and display a CD62L^low^CD69+ phenotype^5,8,11^. Importantly T_RM_ have recently been recognized as the predominant surveyor of NLT^5,8,12^ where they provide potent front-line defense against pathogen reinfection^13–15^. CD8+ T_RM_ and CD8+ T cells with T_RM_ phenotype have been identified in tumors and associated with improved prognosis^16–18^. Recent studies using T cell receptor transgenic T cells to generate skin T_RM_ have identified a role for these cells in localized immunosurveillance that promotes protection from melanoma^19,20^, and have revealed functional relationships between CD8+ T cell subsets during anti-tumor responses^21^.

Therapeutic efficacy and patient responsiveness to ICI therapy are strongly linked to T-cell mediated immune responses, and positively correlated with the availability of tumor-derived neoantigens^22–24^. Mutation-dense or DNA mismatch repair-deficient cancers such as squamous carcinomas and melanoma typically evoke stronger CD8+ T cell responses to ICI therapy, compared to more genetically stable carcinomas such as pancreatic and urothelial^25,26^. Thus, particularly in the case of cancers where tumor antigen density and/or T cell infiltration is low, local sensing and presentation of tumor neoantigens via dendritic cells to T cells is a critical rate-limiting step in driving host-mediated CD8+ immunity to neoplastic cells^4^. Localized physical destruction or ablation of tumors in vivo has been proposed to aid in tumor clearance. This likely occurs by liberating tumor antigen and facilitating antigen presentation that primes T cells, thereby functioning as an in situ vaccination which ultimately induces long-term immune protection^27–30^. These approaches are also predicted to synergize with checkpoint therapy either directly, by potentiating the effects of anti-CTLA-4 on sustaining T cell priming, or indirectly, by promoting the expansion of pre-existing tumor-specific T cells that can be targeted for increased intratumoral functionality by anti-PD-1/PD-L1 inhibitors^3^. A wide range of focal tumor destruction methods, including radiation, thermal ablation (radiofrequency or microwave), cryotherapy, and high intensity focused ultrasound, have been hypothesized to release tumor antigen to promote CD8+ T cell activation^31–33^. However, few studies have closely monitored endogenous tumor-antigen-specific CD8+ T cells following combinatorial ICI therapy and in situ tumor destruction. One study using the transplantable TRAMP-C2 mouse prostate carcinoma model^34^ showed that cryotherapy in combination with anti-CTLA-4 ICI was associated with reduced tumor burden during a second tumor re-challenge^35^. This effect was correlated with increases in the fraction of CD8+ T cells specific for the prostate tumor antigen SPAS-1 (stimulator of prostatic adenocarcinoma-specific T cells/SH3GLB2), for which a neoantigen point mutation has been identified in TRAMP-C2 cells^36^.

Recently, a soft tissue ablation technique known as irreversible electroporation (IRE) has been developed that uses electrical pulses to disrupt cell membranes^37^. Due to this controlled and non-thermal energy delivery mechanism vascular tissue can be preserved during IRE treatment, making it potentially useful for ablating complex tumor-organ environments^37–39^. Several clinical trials, applied to pancreatic, prostate, liver, and kidney tumors indicate that IRE is associated with varying degrees of clinical success^40–44^, and recent studies in both humans and mice suggest an overall increase in tumor infiltration of bulk CD8+ T cells^45–47^. Moreover, the transient increase of tumor-infiltrating CD8+ T cells after IRE is usually accompanied by immune suppression^41,42,48,49^. Immunomodulatory approaches, including GM-CSF^50^ and STING agonist^51^, improve the overall immune response following IRE. Most recently, ICI has been demonstrated to significantly improve systemic immune response in IRE treating preclinical pancreatic cancer^52,53^, leading to a clinical trial with IRE and the anti-PD-1 ICI Nivolumab (NCT03080974). However, detailed analysis of tumor neoantigen-specific immunological responses that provide insight into the mechanism of action of IRE as an in situ vaccination is limited^42,54^. Using an in vitro system, we recently demonstrated that IRE leads to enhanced tumor antigen release and presentation compared to other modes of focal tumor destruction^55^, but to date, there are limited mechanistic information available linking primary endogenous T cell responses in vivo to combined IRE and immunotherapy strategies. Furthermore, the dynamics and fate of tumor antigen-specific—and in particular tumor-specific CD8+ T_RM_ cells—during interventions that combine immunotherapy with in situ ablation therapy such as IRE have not been explored.

In this work, we test the hypothesis that primary tumor destruction by IRE in combination with ICI will increase systemic and T_RM_ tumor-specific memory T cells and promote tumor clearance. We demonstrate that endogenous CD8+ T cells specific for the SPAS-1 neoantigen expand in number and are enriched in tumors following challenge with TRAMP-C2 tumor cells, and give rise to CD8+ T_RM_ cells in diverse tissues. Combining IRE with anti-CTLA-4 promotes a systemic wave of functional neoantigen-specific T cells that is associated with increased primary tumor regression, deposition of tumor-specific T_RM_ cells in multiple NLTs, and protection from tumor re-challenge. Dual therapy also sensitizes refractory tumors to subsequent therapy with anti-PD-1, providing preclinical evidence for the rational and temporal application of multiple physical and ICI tumor therapies.

## Results

### Quantification of SPAS-1 specific T cell expansion

**Fig. 1:**
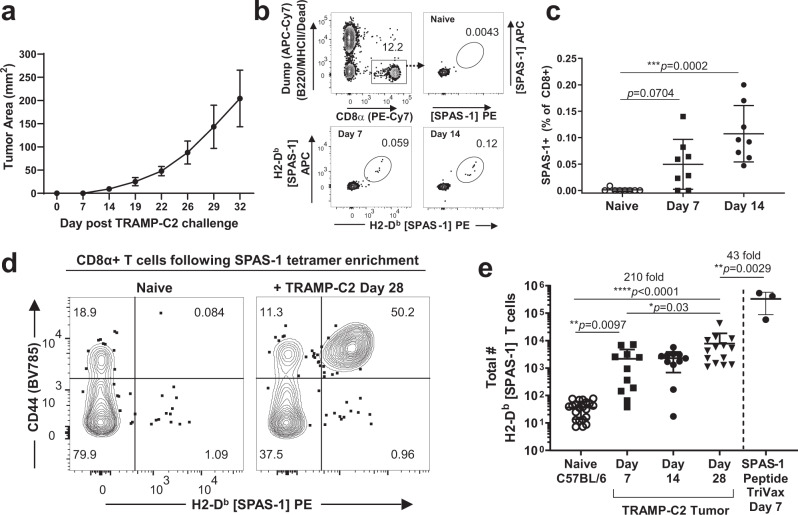
Quantification of SPAS-1 neoantigen-specific CD8+ T cells following TRAMP-C2 tumor challenge. **a** TRAMP-C2 cells were injected s.c. in the flank of C57BL/6J mice and tumor growth measured. Average of four mice from one representative experiment of four performed. **b** Representative flow cytometry staining of blood collected from mice before (naive) and at the indicated times after TRAMP-C2 tumor challenge. SPAS-1+ T cells within the CD8+ T cell gate were identified by dual staining with phycoerythrin (PE)- and allophycocanin (APC)- labeled peptide-MHC-I tetramers loaded with STHVNHLHC peptide specific for the H8 neoantigen of SPAS-1 in TRAMP-C2 cells. **c** Quantification of staining shown in (**b**), compiled in multiple mice. Kruskal–Wallace test was performed, *p* = 0.0005, with exact *p* values by Dunn’s multiple comparision test; ***p < 0.001. Results in (**a**–**c**) are representative of four independent experiments performed. (**d**, **e**) Single-cell suspensions of pooled spleen and peripheral lymph nodes from naive tumor-bearing mice were incubated with PE- and APC- fluorochrome tetramerized H2-D^b^ SPAS-1. Tetramer-binding cells were captured by enrichment over anti-flurochrome magnetic beads and then stained for flow cytometry. **d** Representative flow cytometry staining of gated CD8+ T cells after tetramer enrichment showing expression of PE-labeled tetramer and CD44. **e** Quantified and pooled data showing values for individual naive (*n* = 21) or TRAMP-C2 challenged mice at days 7, 14, and 28 (*n* = 11, 11, and 14, respectively). Data are derived from 5, 3, and 4 independent experiments, respectively, for naive, day 7 and 14, and day 28 following tumor challenge. For SPAS-1 tetramer enrichment from naive mice injected i.v. with the SPAS-1 peptide in the presence of Poly I:C and anti-CD40 (TriVax), one representative experiment with three biological replicates is shown, of two similar experiments performed. Kruskal–Wallace test was performed, *p* < 0.0001, with exact *p* values by Dunn’s multiple comparison test; *p < 0.05, **p < 0.001, ****p < 0.0001. Bars represent mean ± S.E.M. Source data are provided as a Source Data File.

To aid in monitoring systemic immune responses to cancer immunotherapy, we first characterized the endogenous CD8+ T cell response to syngeneic TRAMP-C2 tumor injected subcutaneously into C57BL/6 mice. Tumor growth was detected ~2 weeks after injection and mice were maintained for at least 5 weeks with this relatively slow-growing tumor (Fig. 1a). An immunodominant mutation in the SPAS-1 protein was previously identified in this cell line, facilitating detection of tumor neoantigen-specific CD8+ T cells using MHC-I H-2D^b^ tetramers loaded with the STHVNHLHC (H8) peptide^36^. Sampling of blood in the days following tumor implantation revealed a small population of CD8+ T cells that are recognized by the fluorescent SPAS-1 tetramer reagent (Fig. 1b). These expanded cells typically represented between 0.05 and 0.2% of the CD8+ T cells in the blood (Fig. 1c). To directly enumerate the total SPAS-1-specific T cell response in the spleen and lymph nodes of TRAMP-C2 bearing mice, we adopted a magnetic enrichment technique originally used in infection settings^56^. SPAS-1+ CD8+ T cells were rare in pooled lymph nodes and spleen from unchallenged mice (Fig. 1d, left). The captured SPAS-1+ cells expressed low levels of the glycoprotein CD44, consistent with a naive phenotype^9^ (Fig. 1d). These were enumerated between 7 and 77 per mouse, with a calculated average naive precursor frequency of 37 total SPAS-1 specific CD8+ T cells per mouse (Fig. 1e). Following tumor challenge, a large population of tetramer-binding SPAS-1+ CD8+ T cells was readily captured (Fig. 1d). These cells expressed higher levels of CD44, indicative of prior antigen experience (Fig. 1d). Numerically, there was a 210-fold expansion of SPAS-1 specific CD8+ T cells after tumor challenge yielding an average of 7832 CD8+ T cells in the lymphoid organs of a single mouse (Fig. 1e). This expansion was not an upper limit however, as the lymphoid organs of naive mice immunized intravenously with SPAS-1 (H8) peptide in a TriVax formulation containing anti-CD40 and Poly I:C^57^ accumulated over 40-fold more SPAS-1 specific T cells than were generated by the tumor response (Fig. 1e).

### SPAS-1 tumor-specific CD8+ T cells accumulate in NLT and are tissue resident

**Fig. 2:**
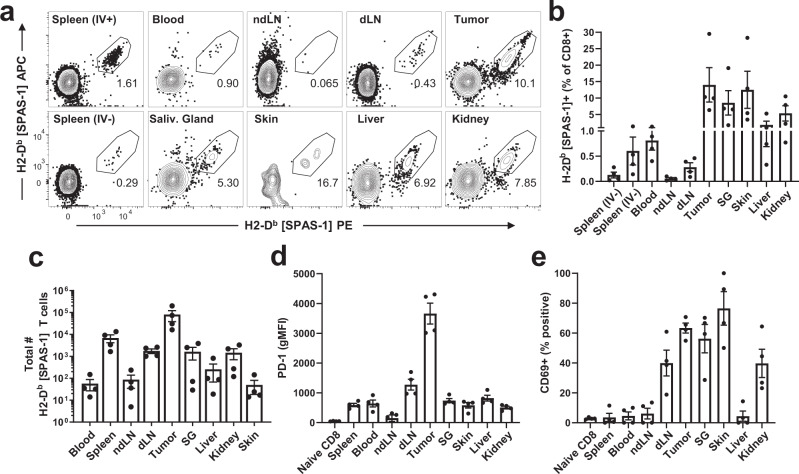
SPAS-1+ T cells are broadly distributed in lymphoid and non-lymphoid tissues following TRAMP-C2 tumor challenge. Mice were challenged s.c. with TRAMP-C2 in the flank and necropsies were performed 28 days later. Mice were injected i.v. with 3 µg of FITC-labeled anti-CD8α antibody 3 min prior to harvest, to identify CD8+ T cells in the circulation. Single-cell suspensions from the indicated organs were prepared (see Methods) and stained for flow cytometry. **a** Representative FACS plots showing dual fluorescent staining for SPAS-1 tetramer within the CD8+ IV- gate (see Supplementary Fig. 1a, b). **b**, **c** Quantification of replicate mice showing the frequency and total number of SPAS-1+ T cells. **d**–**e** Expression of PD-1 and CD69, respectively, on gated IV- SPAS-1+ T cells. See also Supplementary Fig. 1c. Results in (**b**–**e**) represent four biologically independent replicates from three independent experiments assessing SPAS-1+ CD8+ T cells in these tissues. Bars represent mean ± S.E.M. IV intravascular (− or +), SG salivary gland, dLN tumor-draining lymph node, ndLN non-draining LN. Source data are provided as a Source Data File.

We surveyed several non-lymphoid organs to assess the distribution of SPAS-1 specific CD8+ T cells localized outside of the bloodstream and lymphoid tissues, 28 days after tumors were established (Supplementary Fig. 1a). As expected, tetramer-binding T cells were concentrated in the tumor site (Fig. 2a–c). In contrast to the spleen, blood, and non-draining lymph nodes, SPAS-1 T cells in the tumor expressed high levels of PD-1 (Fig. 2d, left, and Supplementary Fig. 1c). We also detected SPAS-1 specific T cells in several NLTs including liver, lung, kidney, salivary gland, and skin (Fig. 2a–c, Supplementary Fig. 1b). Specificity of the tetramer is indicated by staining of these NLT in control single-cell suspensions generated from age-matched naive mice not challenged with TRAMP-C2 cells (Supplementary Fig. 1d) and with non-cognate tetramer control staining of tissues (Supplementary Fig. 1d, right) and TRAMP-C2 tumors (Supplementary Fig. 1e). Tumor-induced SPAS-1 specific T cells in NLTs adopted phenotypes consistent with a tissue-resident population including expression of CD69 (Fig. 2e and Supplementary Fig. 1c, right). We also noted that significant portions of these populations of SPAS-1 specific T cells were outside of blood vessels based on their exclusion from intravascular staining (IV-neg) by injection of anti-CD8α antibody three minutes prior to tissue harvest^11^ (Supplementary Fig. 1b).

**Fig. 3:**
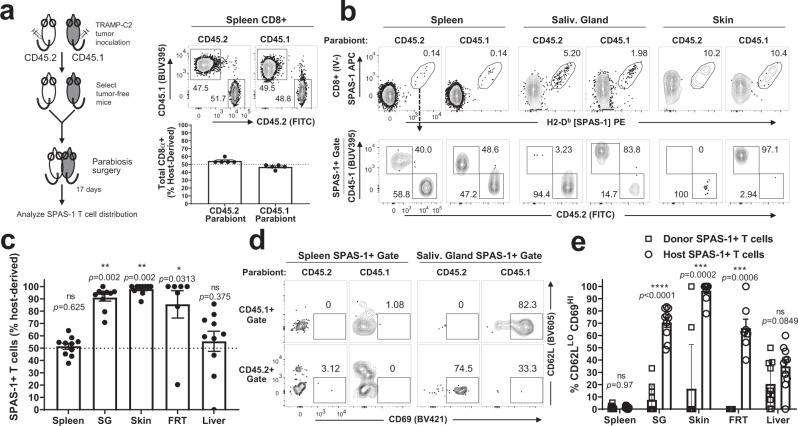
SPAS-1+ memory T cells in non-lymphoid tissues are tissue resident. Female CD45.2 and CD45.1 mice were challenged s.c. in opposite flanks with TRAMP-C2 tumor cells. Mice lacking tumors 6 weeks later but exhibiting residual SPAS-1+ CD8+ memory T cell response in the blood were selected. **a** Schematic of experimental design. Parabiosis was performed to surgically join the circulation of challenged but tumor-free CD45.2 and CD45.1 mice. After 17 days of equilibration, mice in each pair were individually injected i.v. with 3 µg of FITC-labeled anti-CD8α antibody 3 min prior to harvest, to mark CD8+ T cells in the circulation. Single-cell suspensions from the indicated organs were prepared and stained for flow cytometry. CD8+ T cells from the spleen of one parabiotic pair are discriminated with antibodies to CD45.2 and CD45.1, quantified in the bottom graph. Data points in (**a**), (**c**), (**e**) reflect individual biological replicate mouse pairs (five pairs total), from one of two similar experiments performed. **b** Representative flow plots visualizing three different tissues of a single parabiotic pair. Top row, SPAS-1+ gate in CD8+ IV- T cells. Bottom row, CD45.2, and CD45.1 staining of SPAS-1+ T cells from the plot above. **c** Quantification of data from five parabiotic pairs, calculating the proportion of SPAS-1+ T cells in each tissue that was derived from the host mouse as fraction of the total from both host and donor mouse. Significance was determined by two-sided Wilcoxon sign-rank test with a theoretical value of 50; *p < 0.05; **p < 0.01. **d** Representative flow plots showing the expression of CD62L and CD69 cell surface phenotype of IV- SPAS-1+ T cells from each partner, separated by partner. **e** Quantification of the fraction of CD62L^lo^CD69^hi^ SPAS-1 T cells in each mouse, derived from the host or donor partner. Unpaired two-sided Mann–Whitney test was performed; ***p < 0.001; ****p < 0.0001. All error bars represent mean ± S.E.M. IV intravascular, SG salivary gland, FRT female reproductive tract. Source data are provided as a Source Data File.

Next, we used parabiosis to formally test whether the endogenously expanded SPAS-1+ T cells that accumulated in NLTs were tissue-resident. This surgical approach joins the vasculatures of two mice and allows distinction between cells that can freely circulate between the parabiont partners and cells that remain resident in the original host^8^. Since not all mice challenged with TRAMP-C2 tumor cells develop a physical tumor, tumor-free mice with detectable SPAS-1 T cells in the blood were selected. Age-matched but congenically mis-matched CD45.2 and CD45.1 mice were surgically paired, and antibodies specific for either CD45 allele were used to distinguish the host of origin (Fig. 3a, left). After 17 days, near 50:50 equilibration of total CD8+ T cells was observed in the spleens of the CD45.1 and CD45.2 parabionts (Fig. 3a–c, spleen). By contrast, we observed strong disequilibrium of SPAS-1+ CD8+ T cells in several NLTs (Fig. 3b, c). In accordance with the parabiosis migration data, the host-biased tumor-specific T cells were predominantly IV-neg and displayed a CD62L^lo^CD69^hi^ phenotype consistent with tissue residency (Fig. 3d, e). Rare SPAS-1+ CD8+ T cells from the donor parabiont accessed tissues in the host parabiont; however, these immigrant cells displayed significantly decreased expression of tissue residency markers (Fig. 3d, e). Together these data suggest that the majority of host SPAS-1+ memory CD8+ T cells in the NLTs we tested are bona fide non-recirculating tissue-resident memory T cells.

### ICI fails to control tumor growth and does not alter SPAS-1 T cell distribution

Despite expansion and persistence of the SPAS-1+ memory CD8+ T cell population in tumors and NLTs, established TRAMP-C2 tumors were not spontaneously cleared from mice (Fig. 1a), consistent with previous observations^35,58^. We observed that monotherapy with ICI antibodies targeting PD-1, PD-L1, and CTLA-4 failed to impede tumor growth when applied to tumor-bearing mice (Supplementary Fig. 2a). There was also no impact of anti-PD-1, anti-PD-L1, or anti-CTLA-4 ICI therapy on the frequency of CD8+ SPAS-1+ T cells in tumor following any of the ICI therapy regimens (Supplementary Fig. 2b). Furthermore, anti-CTLA-4 monotherapy did not affect the number of SPAS+ T cells in the spleen, tumor, or NLT at the experimental tumor endpoint (Supplementary Fig. 2c). Together, these observations indicate that ICI monotherapy on the established tumor was insufficient to substantially alter tumor growth or antigen-specific T cell responses in this tumor model.

### IRE augments anti-CTLA-4 therapy and improves tumor outcome

**Fig. 4:**
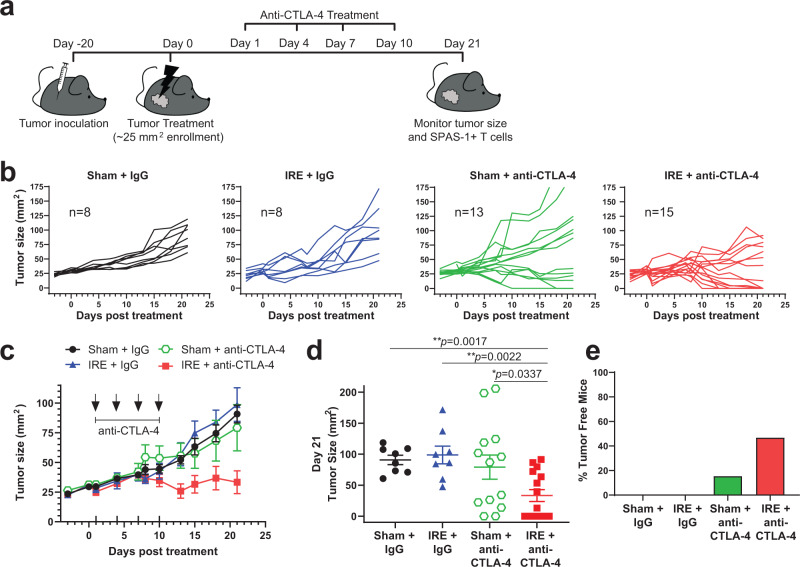
Irreversible electroporation augments anti-CTLA-4 therapy and improves tumor outcome. **a** Schematic of experimental design. Briefly, mice were injected intradermal (i.d.) with 1.0 × 10^6^ TRAMP-C2 cells into the right flank, and animals were enrolled into the indicated treatment group when their tumor reached 4–5 mm in diameter. **b** Individual tumor growth curves following each treatment, with the time synchronized to treatment on day 0. **c** Averaged tumor growth curves from (**b**). **d** Individual tumor sizes on day 21 from each treatment group in (**b**). **e** Fraction of tumor-free mice from each group, at day 21 post-treatment. Data in (**b**–**e**) reflect *n* = 8–15 mice/group pooled from four independent experiments, with exact n shown on panel **b** Unpaired two-tailed Mann–Whitney test was performed; *p < 0.05; **p < 0.01. Bars represent mean ± S.E.M. Source data are provided as a Source Data File.

We recently reported that tumor cell destruction by IRE is a superior antigen release modality, compared to cryoablation or thermal ablation^55^. Previously published results demonstrated impairment of secondary tumor growth after primary TRAMP-C2 tumors were destroyed by cryotherapy combined with anti-CTLA-4 treatment^35^. We sought to test whether local tumor destruction by IRE followed by anti-CTLA-4 therapy would also perform an in situ vaccination function. Specifically, we tested whether IRE in combination with anti-CTLA-4 would promote SPAS-1+ CD8+ T cell expansion and regression of residual primary tumor growth in the TRAMP-C2 model (Fig. 4a). We used a parallel plate electrode to treat 5 mm diameter tumors by IRE at a reduced dose chosen to remove ~50% of tumor tissue. Thus, mice treated with IRE maintained a fraction of residual or undertreated tumor that could be subsequently monitored for continued growth or remission, and recapitulates a common limitation of the inability to eliminate 100% of the tumor. In comparison to sham-treated tumors, some IRE-treated tumors showed a brief delay of growth kinetics, although associated edema and swelling from the procedure masked temporary differences in tumor lesion size (Fig. 4b). Over a 21-day period following treatment, 100% (8/8) of IRE monotherapy tumors re-grew to a size similar to that of sham-treated mice or reached tumor endpoint criteria (Fig. 4a–d).

We next tested if anti-CTLA-4 ICI therapy in combination with IRE could improve tumor control. Anti-CTLA-4 was injected once every 3 days for four total doses, beginning the day following IRE treatment, and tumor outgrowth was monitored. Sham treatment followed by anti-CTLA-4 (Sham + anti-CTLA-4) generally failed to impede tumor growth, as continued tumor growth was observed in more than half (53%; 7/13) of mice with this treatment arm (Fig. 4b–d). A small subset (2/13) of mice in this cohort eliminated the tumor (Fig. 4b, d, e), although this response was not always observed (see Supplementary Fig. 2). By contrast, a combinatorial regimen of IRE followed by anti-CTLA-4 therapy led to complete tumor regression in nearly half (46%; 7/15) of mice (Fig. 4b, e). Dual therapy was also associated with an overall decrease in average tumor size over time (Fig. 4c) and significantly reduced tumor burden at day 21 (Fig. 4d). In summary, dual therapy with IRE plus anti-CTLA-4 provide a significant therapeutic advantage over either IRE, or anti-CTLA-4 monotherapy alone.

### Combination IRE and anti-CTLA-4 therapy increases intratumoral SPAS-1+ T cells

**Fig. 5:**
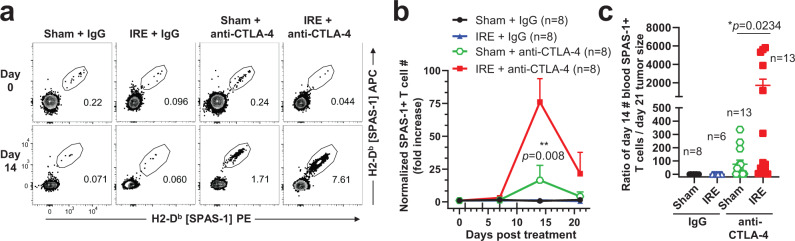
SPAS-1+ T cells are increased in the blood following combination therapy and predict tumor outcome. Mice bearing TRAMP-C2 tumors were treated as in Fig. 4. **a** Representative SPAS-1 tetramer staining of CD8+ T cells in peripheral blood at days 0 and 14 following the indicated treatment. **b** The absolute number of SPAS-1+ T cells per 100 µl of blood was calculated at days 7, 14, and 21 following treatment and normalized to the baseline in each mouse at day 0. **c** Ratio of the number of SPAS-1+ T cells per 100 µl of the blood of each mouse at day 14 over the endpoint tumor size at day 21. Data in (**b**) represent blood tested in *n* = 8 individual mice/group. **c** *n* = 6–13 individual mice/group, with exact n shown for each group. Data pooled from four independent experiments. Bars represent mean ± S.E.M. Unpaired two-tailed Mann–Whitney test was performed; *p < 0.05, **p < 0.01 Source data are provided as a Source Data File.

To determine whether changes in the number of TRAMP-C2-specific SPAS-1+ CD8+ T cells population were associated with the observed therapeutic efficacy, we first sampled blood as described above (Fig. 1c). Enumeration of SPAS-1+ T cells from quantitative blood draws performed in the same mice before (day 0), during (day 7), and after (day 14) dual therapy revealed a significant amplification peaking 14 days following IRE therapy, compared to other treatment conditions (Fig. 5a). Following dual therapy, the absolute number of SPAS-1+ T cells per blood volume increased nearly 75-fold over baseline values (Fig. 5b). When expressed as a ratio of tumor size on day 21 to T cell number in the blood on day 14, the therapy-mediated boost in peripheral T cell numbers were negatively correlated with sizes of tumor at day 21 (Fig. 5c).

**Fig. 6:**
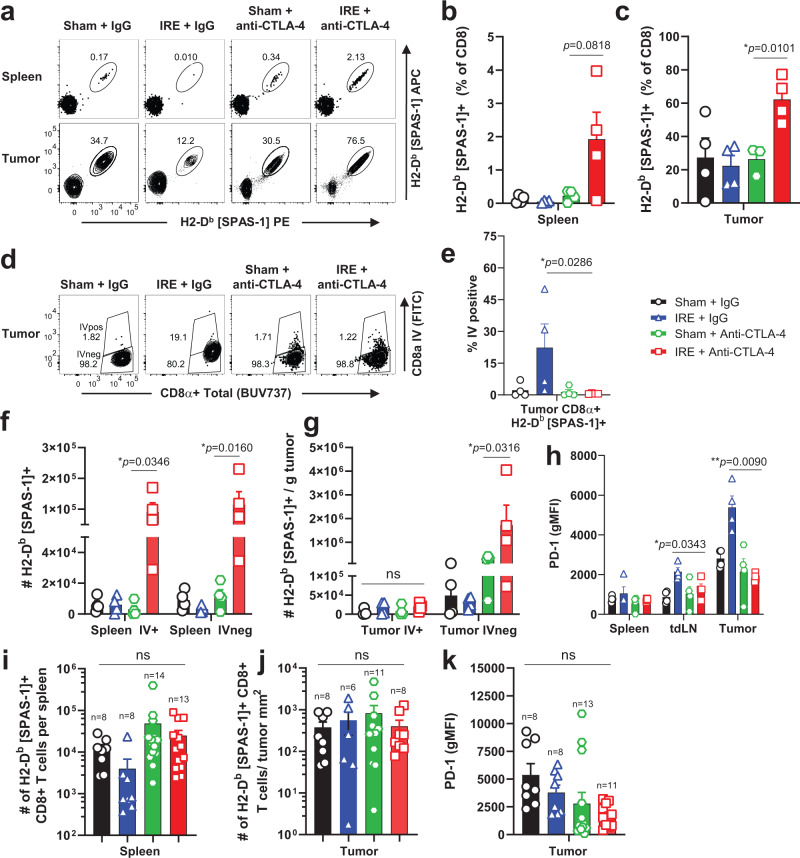
Combination therapy is associated with increased numbers of tumor-specific SPAS-1+ T cells in spleen and tumors. Mice bearing ~4–5 mm diameter TRAMP-C2 tumors were treated with Sham, IRE, and/or anti-CTLA-4 as in Fig. 4. On day 14 (**a**–**h**) or day 21 (**i**–**k**) following therapy, single-cell suspensions from the indicated tissues were analyzed by flow cytometry. Data in (**b**–**h**) represent individual mice (*n* = 4/group) pooled from two independent experiments, and **i**–**k** represent individual mice (*n* = 6–14/group) pooled from four independent experiments, with exact n shown for each group. **a** Representative tetramer staining of SPAS-1 from spleen and tumor samples in each treatment group, gated on Live, Thy1.2+ CD8+ T cells. **b**, **c** Frequency of SPAS-1+ T cells in spleen and tumor, respectively. Intravascular (IV) anti-CD8α was injected 3 min prior to sacrifice. **d**, **e** Representative flow cytometry plots and quantification, respectively, gated on Thy1.2+ CD8 + SPAS-1+ T cells, showing IV CD8α stain used to segregate the IV-negative and -positive fractions from each treatment group. **f**–**g** Total number of SPAS-1+ CD8+ T cells in the indicated IV fraction of spleen and tumor, respectively. **h**, **k** PD-1 expression at days 14 and 21, respectively. **i**–**j** Total number of SPAS-1+ T cells in the IV-negative fractions of spleen and tumor, respectively at day 21 following the start of therapy. Bars represent mean ± S.E.M. **b**, **c**, **e**–**k**, Unpaired two-tailed *T* test with Holm-Sidak’s correction was performed; ns, not significant, *p < 0.05, **p < 0.01. Source data are provided as a Source Data File.

Consistent with the timing of peak SPAS-1+ response in blood, the frequency of SPAS-1+ cells was also amplified at day 14 in both the spleen (Fig. 6a, b) and tumor (Fig. 6a, c) of dual therapy mice. The absolute number of SPAS-1+ CD8+ T cells was also increased in the spleen (Fig. 6f) and tumor (Fig. 6g) at this time point. The numerical increase was apparent in both the spleen red pulp (IV-pos) and white pulp (IV-neg) populations. Analysis of intravascular labeling also allowed discrimination of T cells in the tumor tissue itself, versus those circulating through or trapped in the tumor vasculature, at the moment of sacrifice (Fig. 6d). This revealed that the majority (>95%) of these expanded SPAS-1+ T cells in the IRE+ anti-CTLA-4 condition were in the tumor IV-neg gate and therefore concentrated in the tumor (Fig. 6d, e, g). By contrast, there was no difference in the absolute number of SPAS-1+ T cells in the tumor vasculature (Fig. 6g, IV-pos). Interestingly, in the IRE-alone condition a subset (~25%) of bulk (SPAS-1 tetramer-negative) CD8+ T cells were labeled with the intravascular CD8α antibody stain (Supplementary Fig. 3a, b) indicating slightly reduced access of these CD8+ T cells to the parenchyma of tumors following IRE monotherapy treatment. A similar trend of exclusion from the tumor was observed for SPAS-1 tetramer-positive cells in tumors from mice treated with IRE monotherapy (Fig. 6d, e).

As the number of SPAS-1+ T cells in blood, spleen, and tumor had contracted to comparable levels and with similar intratumoral PD-1 expression by day 21 (Fig. 5b; Fig. 6i–k), we further examined the intratumoral phenotypes and functional characteristics of SPAS-1+ T cells from dual IRE + anti-CTLA-4 therapy at day 14. PD-1 expression on the tumor-specific T cells following anti-CTLA-4 alone or dual therapy remained slightly elevated in the tumor compared to the spleen 14 days after treatment (Fig. 6h; Supplementary Fig. 4a). However, PD-1 expression was significantly increased on the small population of SPAS-1+ cells in tumors treated with IRE alone, suggesting some level of exhaustion or dysfunction (Fig. 6h; Supplementary Fig. 4a). Indeed, expression of the inhibitory receptors LAG-3 and TIM-3 was also elevated on SPAS-1+ T cells after IRE monotherapy (Supplementary Fig. 4b, c), compared to the expanded population of intratumoral T cells following IRE + anti-CTLA-4 dual therapy. Similarly, expression of the exhaustion-associated transcription factor TOX was significantly elevated in the draining lymph node and tumor of IRE-treated mice but this increase was not observed following anti-CTLA-4 treatment (Supplementary Fig. 4d).

To test functionality of the tumor-specific T cells, we examined the IFN-gamma and TNF-α cytokine secretion capacity by restimulating SPAS-1+ CD8+ T cells on day 14 following each treatment condition. Under most conditions, cytokine production was similar between spleen and tumor, indicating high potential functionality, with ~75% of cells secreting IFN-gamma and ~nearly 70% producing TNF-α (Supplementary Fig. 5a–c). However, tumor-specific T cells from IRE monotherapy showed a trend of suppressed cytokine production, especially when assessed for capacity to secrete both cytokines (Supplementary Fig. 5a, d). Of note, cytokine production by tetramer-negative (non-SPAS-1+) CD8+ T cells was also enriched and elevated in tumors from all treatment groups (Supplementary Fig. 3d) indicating that in addition to SPAS-1, there are T cells of diverse specificity involved in the overall CD8+ T cell response to TRAMP-C2 tumors. The overall number of bulk CD8+ T cells in tumors increased following anti-CTLA-4 treatment alone or in combination with IRE (Supplementary Fig. 3c), prompting us to investigate whether previously reported reductions in regulatory T cells (Treg) were potentially involved^59^. However, no change in the frequency or number of CD4+ FoxP3+ T cells was observed at the day 21 tumor endpoint (Supplementary Fig. 6a–c) or at the day 14 peak response (Supplementary Fig. 6d, e) following anti-CTLA-4 treatment, except for a trend of increased frequency of Treg cells in the IRE monotherapy group that was not translated to differences in total numbers due to reduced overall numbers of CD4 T cells.

Given the increase in tumor parenchyma-infiltrating SPAS-1+ CD8+ T cells in refractory tumors following combination IRE + anti-CTLA-4 therapy, we also wondered if these tumor-specific T cells adopted characteristics of tissue-resident memory cells. As in Fig. 2b, staining for CD69 was upregulated on the majority of intratumoral (IVneg) SPAS-1+ T cells at both days 14 and 21 (Supplementary Fig. 7a–c). Expression of canonical tissue residency markers, including the epithelial cadherin-binding integrin CD103 and the collagen-binding integrin CD49a, was less prominent, with <20–25% expressing CD103, and variable expression of CD49a on tumor-infiltrating T cells (Supplementary Fig. 7a, d–f). Furthermore, we did not observe differences in these tissue residency markers compared between treatment groups at either time point. These data are consistent with the possibility that a subset of tumor-infiltrating SPAS-1+ T cells are resident in the tumor, but additional characterization would be required to confirm intratumoral tissue-residency status.

### Combination IRE and anti-CTLA-4 therapy increases SPAS-1+ T_RM_ in NLT

**Fig. 7:**
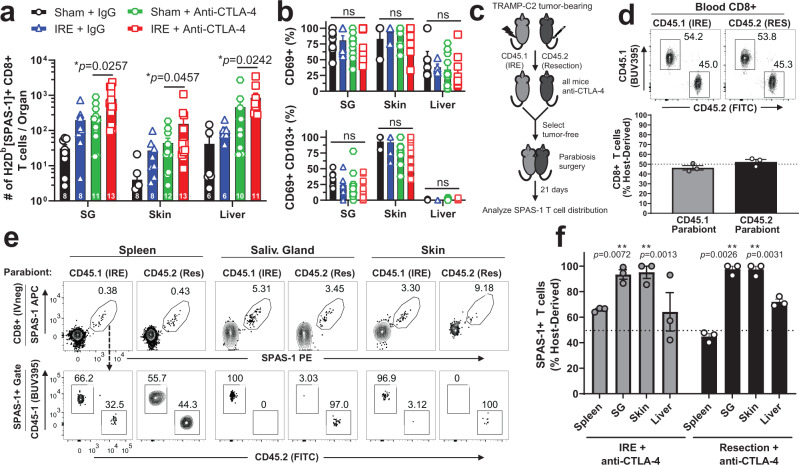
Combination therapy is associated with increased numbers of tissue-resident memory SPAS-1+ T cells in non-lymphoid tissues. Mice bearing TRAMP-C2 tumors were treated as in Fig. 4. **a** 21 days following therapy, the number of SPAS-1 + T cells was quantified by flow cytometry from the indicated tissues. **b** Percentage of SPAS-1+ T cells identified in (**a**) that express CD69 (top panel) or CD69 and CD103 (bottom panel). Data in (**a**, **b**) represent individual mice (*n* = 8–13/group) pooled from four independent experiments, with exact n for each group shown at the base of each bar for each tissue. The exact n values for the number of samples analyzed in (**b**) are shown in the Source Data File. **c** TRAMP-C2 tumors were inoculated i.d. into CD45.1 and CD45.2 mice and tumors were removed by IRE or surgical resection, respectively, followed by anti-CTLA-4, every third day for four total doses. Mice that cleared tumors were selected and rested for three additional weeks, and parabiosis surgery was performed. **d** Flow cytometry of blood collected from each partner mouse 17 days after surgery, showing the fraction of host-derived total CD8+ cells from each parabiont in a representative FACS plot (top) and quantified for 3 pairs of mice (bottom). **e** After 21 days of equilibration, mice in each pair were individually injected i.v. with 3 µg of FITC-labeled anti-CD8α antibody 3 min prior to harvest, to mark CD8+ T cells in the circulation. Single-cell suspensions from the indicated organs were prepared and stained for flow cytometry. Top row, SPAS-1+ T cell gating from the IV-CD8+ T cell population from the indicated organs of each congenically distinct partner is shown. Bottom row, SPAS-1+ T cells from each partner are discriminated by CD45.1 and CD45.2 to determine the partner of origin. **f** Quantification of data from 3 parabiotic pairs, separated by treatment group. The proportion of SPAS-1+ T cells in each tissue that were derived from the host mouse is presented as a fraction of the total from both host and donor mice. **a**–**b** Data pooled from four independent experiments. Unpaired two-tailed Mann–Whitney test was performed; *p < 0.05. **c**–**f** Data representative of one experiment with 3 parabiotic pairs. One sample *T* test with a hypothetical value of 50 was performed; ns, not significant; **p < 0.01. All bars represent mean ± S.E.M. SG salivary gland. Source data are provided as a Source Data File.

We next investigated if the expansion of systemic SPAS-1+ CD8+ T cells was concomitant with increases of non-recirculating SPAS-1+ T cells in NLT distal to the tumor, specifically within the salivary gland, contralateral skin, and liver. Tumor neoantigen-specific SPAS-1+ T cells were significantly increased in each organ following dual therapy, when compared to anti-CTLA-4 monotherapy treatment (Fig. 7a). As these T cells also expressed CD69 and, in some cases CD103 (Fig. 7b; Supplementary Fig. 7a) surface markers consistent with tissue residency, we wanted to formally test if this increased population of cells was tissue-resident. To this end, we performed parabiosis surgery on tumor-free animals that had undergone therapy with either surgical tumor resection or IRE ablation to remove the tumor mass, followed in both cases by treatment with anti-CTLA-4 (Fig. 7c). After equilibration of blood (Fig. 7d), SPAS-1+ T cells isolated from salivary gland, contralateral skin, and liver showed a profound host bias (Fig. 7e, f), similar to that observed above (Fig. 3c). For example, over 90% of all tumor-specific T cells isolated in the SG were bona fide tissue-resident memory T cells. Thus, we concluded that dual therapy significantly amplified the number of SPAS-1+ CD8+ T cells in all NLTs assayed compared to mice that did not receive IRE. Taken together our data suggest tumor control after dual therapy is associated with increased frequency of SPAS-1-specific CD8+ T cells in circulation as well as in NLTs.

### Endogenous tumor antigen-specific T_RM_ associated with tumor protection

**Fig. 8:**
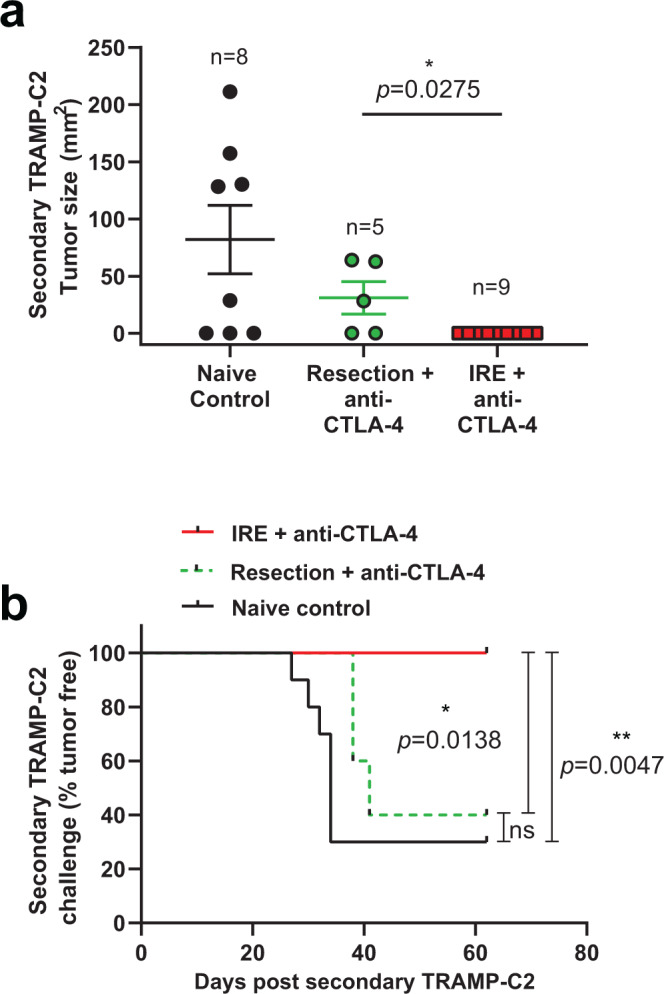
IRE plus anti-CTLA-4 combination therapy is associated with protection from secondary tumor challenge. TRAMP-C2 tumors were inoculated into the right flank of C57BL/6J mice and allowed to grow to ~4–5 mm diameter. Tumors were removed by IRE or surgical resection, followed by treatment with four doses of anti-CTLA-4. Tumor-free mice were selected and rested for three additional weeks when mice were re-challenged in the left flank with 1.0 × 10^6^ TRAMP-C2 cells. **a** Tumor size 42 days after secondary tumor challenge, with exact n values shown and data points reflecting independent biological replicate mice. Age-matched naive mice were used as a control, demonstrating typical ~60–70% tumor “take” rate. Bars represent mean ± SEM. Unpaired two-tailed Mann–Whitney test was performed; *p < 0.05. **b** Kaplan–Meier curves from the mice in (**a**), noting the day of tumor incidence following secondary tumor challenge. Two-sided Mantel-Cox log rank test was performed; *p < 0.05; **p < 0.01. Source data are provided as a Source Data File.

Tissue-resident memory T cells have been implicated in tumor protection, although few studies to date have formally tested the function of endogenously expanded tumor antigen-specific memory T cells. Our dual therapy promoted an expanded population of these cells by day 21 in several NLTs including the skin (Fig. 7a), but equivalent numbers in the blood circulation at this time point (Fig. 5a, b). We therefore hypothesized that the presence of expanded tumor-specific T cells in NLTs would be associated with enhanced protection from secondary tumor challenge at a skin site distal from the primary tumor challenge. We generated a cohort of mice that had fully cleared primary tumor either through dual IRE + anti-CTLA-4 therapy, or from resection plus anti-CTLA-4 therapy as a control. Mice were rested for at least 21 days between the last detectable tumor growth and the secondary tumor challenge in the skin on the opposite flank. TRAMP-C2 tumors grew in age-matched naive mice with a typical 60–70% take rate (Fig. 8a). A similar fraction of mice that received resection + anti-CTLA-4 therapy also developed tumors although the tumor sizes trended smaller (Fig. 8a). By contrast, mice that had previously achieved complete remission following dual IRE + anti-CTLA-4 therapy were 100% protected from secondary tumor challenge, and showed significant survival advantage over mice not receiving IRE therapy (Fig. 8b, 9/9 mice).

### Secondary therapy with anti-PD-1 augments IRE/anti-CTLA-4 combination

**Fig. 9:**
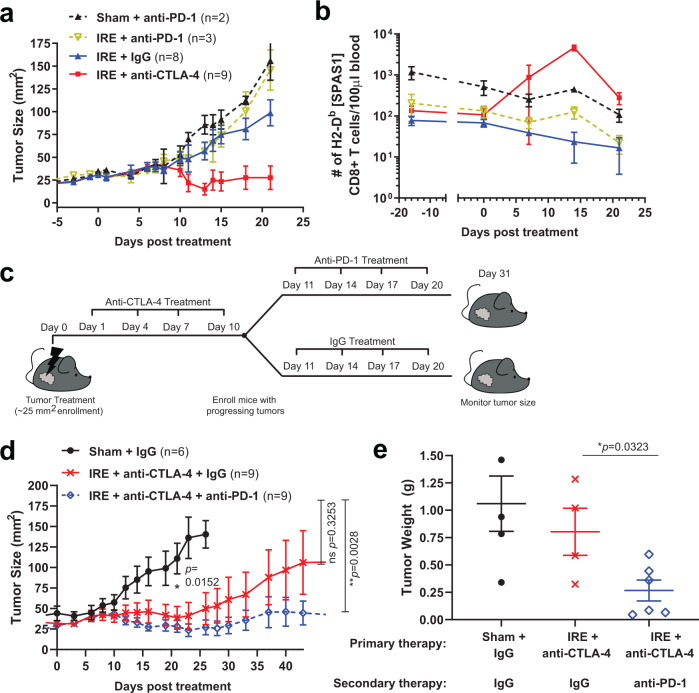
Anti-PD-1 treatment following IRE plus anti-CTLA-4 combination therapy sustains tumor regression. **a** Mice bearing TRAMP-C2 tumors were treated with IRE or sham, followed by either IgG, anti-CTLA-4 or anti-PD-1 as indicated. Average tumor growth is shown. **b** The absolute number of SPAS-1+ T cells per 100 µl of blood was determined by flow cytometry at days 0, 7, 14, and 21 following treatment in **a**. Data in (**a**) reflects 2–9 independent mice treated per group, of one representative experiment performed. Exact n values are shown for each group, with bars representing the mean ± S.E.M. Data in (**b**) reflects the analysis of blood from two independent mice from the experiment in (**a**), depicted as the mean ± the range. **c** Schematic of experimental design. Mice were injected in the flank with TRAMP-C2 cells as in Fig. 4. Tumors reaching 4–5 mm diameter were treated with IRE on day 0, followed by anti-CTLA-4 on days 1, 4, 7, and 10. On day 11, mice were randomly grouped for secondary therapy with 4 doses every 3 days of either IgG (control) or anti-PD-1. **d** Average tumor growth of each treatment arm, reflecting compiled data from *n* = 6–9 mice/group, pooled from two independent experimental cohorts. Unpaired two-tailed Mann–Whitney test was performed; *p < 0.05, **p < 0.01. All individual mice are shown in Supplementary Fig. 8b. **e** Surviving mice from (**d**) were euthanized on day 53 and tumors weighed; *n* = 4 for Sham + IgG and IRE + anti-CTLA-4 + IgG, *n* = 6 for IRE + anti-CTLA-4 + anti-PD-1. Unpaired two-tailed student’s *T* test was performed; *p < 0.05. Bars in (**d**–**e**) re*p*resent mean ± S.E.M. Source data are provided as a Source Data File.

When phenotyping circulating tumor-specific T cells following dual therapy, we observed small but significant increases in the level of PD-1 expression at day 14 following therapy (see also Supplementary Fig. 8a), suggesting that at least some of these circulating T cells have proliferated and/or recently encountered antigen. In addition, PD-1 expression remained elevated on intratumoral tumor-specific T cells at this time point (Fig. 6h; Supplementary Fig. 4a). This observation raised the possibility that treatment with anti-PD-1 in combination with IRE could also improve therapeutic outcomes including tumor remission, especially since a subset of mice in our dual therapy regimen failed to clear tumor (see Fig. 4d, e). However, we were unable to observe an immediate combinatorial effect of anti-PD-1 and IRE on tumor outgrowth (Fig. 9a), and anti-PD-1 with or without IRE treatment did not affect SPAS-1+ T cell levels in the blood (Fig. 9b). Based on the observation that anti-CTLA-4 therapy worked in concert with IRE to boost the number of SPAS-1+ T cells in the blood by day 14 after IRE (Fig. 9b; Fig. 5a, b), we hypothesized that later addition of anti-PD-1 after the conclusion of anti-CTLA-4 therapy, leading up to this numerical peak of the systemic SPAS-1+ T cell response, would further potentiate tumor remission. To perform this study, we enrolled a cohort of mice that had been treated with IRE followed by anti-CTLA-4 on days 1, 4, 7, and 10 post-IRE. Mice were then given 4 doses of either IgG or anti-PD-1 treatment (secondary therapy) over the following 10-day period (days 11, 14, 17, and 20 post-IRE), and tumor growth was tracked >30 additional days (Fig. 9c). Similar to what we observed in Fig. 4c, d, mice were given IRE + anti-CTLA-4 followed by IgG showed overall tumor contraction through day 23, at which time a subset of tumors that were not cleared began to re-grow and ultimately reach tumor endpoints by day ~42 (Fig. 9d and Supplementary Fig. 8b). By contrast, when IRE and anti-CTLA-4 primary therapy was followed up by secondary therapy with anti-PD-1, the average tumor growth was held in check resulting in tumor sizes significantly smaller than the endpoint values of sham-treated tumors (Fig. 9d). Since a subset of mice from either treatment arm cleared tumor by day 23, similar to observations in Fig. 4d, we also retrospectively analyzed growth on the progressing tumors independent of those mice that fully cleared or showed < 9 mm^2^ tumor by day ~23. Exclusion based on these criteria revealed a significant reduction in day 49 tumor size in the secondary PD-1 therapy arm, compared to that of IgG control at day 49 or endpoint (Supplementary Fig. 8c). In addition, remaining tumors in mice surviving until day 53 were excised and weighed at the time of necropsy, and there was a significant decrease in tumor mass in the triple combination therapy group (Fig. 9e). These data indicate that anti-CTLA-4 but not anti-PD-1 impacts tumor growth when applied immediately following IRE, but that synergistic effects of anti-PD-1 can be revealed when dosing is timed to match T cell expansion kinetics.

## Discussion

In this study, we induced durable tumor control and remission by linking the application of ICI and in situ tumor destruction to the mobilization of endogenous tumor antigen-specific T cells and the formation of tissue-resident memory (T_RM_) T cells. We used the transplantable TRAMP-C2 prostate carcinoma cell model, as the rate of tumor growth is slow enough to allow for investigation of memory CD8+ T cells several weeks following tumor inoculation, and the tumors are known to be responsive to ICI treatment^35^. We used MHC-I restricted tetramers recognizing the R/H mutation in the SPAS-1 tumor rejection antigen^36^ to track endogenous CD8+ T cells responding to TRAMP-C2 tumor. Our study expands prior work by combining IRE focal therapy with temporally applied anti-CTLA-4 and anti-PD-1 ICI, and identifies the involvement of the newly defined CD8+ T_RM_ T-cell subset in this immunotherapy.

The small precursor pool of <40 total naive (CD44^lo^) SPAS-1 H8 peptide-specific CD8+ T cells per C57BL/6 mouse is consistent with previous neoantigen repertoire studies^22,60–62^. The absolute size of this population increased substantially following tumor injection, as the TRAMP-C2 tumors expanded. However, this endogenous response was not associated with primary tumor remission, and failed to protect against secondary tumor challenge even when combined with anti-CTLA-4 monotherapy in the absence of tumor destruction (Fig. 7). To further characterize the baseline CD8+ T cell response to tumor challenge, we documented significant numbers of SPAS-1 specific T cells in many non-lymphoid sites, tumor, and SLOs. This wide tissue distribution is consistent with previous reports of pathogen-specific effector and memory T cells^63^, but has not been well described for tumor-specific CD8+ T cells. Many of the SPAS-1+ T cells we identified in NLT adopted surface phenotypes consistent with T_RM_ cells, including CD69, CD49a, and CD103 expression. Intravascular staining indicated that the majority of tumor-specific T cells were excluded from the blood of tumor and NLT sites, indicating concentration in the stroma or parenchyma of tumor or tissue, and consistent with tissue residency. Finally, using parabiosis surgery at memory time points, we confirmed the majority of SPAS-1+ CD8+ T cells in NLT sites were tissue-resident. While bloodborne SPAS-1+ CD8+ T cells could equilibrate through circulation with a conjoined partner, the SPAS-1+ T cells in NLT maintained a profound host bias, indicative of T_RM_.

The induction of pathogen-specific T_RM_ in NLT is a characteristic feature of infection and frequently leads to robust front-line immunity throughout the host^8,13–15,21^. T_RM_ formed by model foreign antigens and/or TCR transgenic cells have been reported in preclinical mouse tumor models, and are associated with prolonged tumor control via promotion of cancer-immune equilibrium^16,19,20^. Parallel phenotypic and transcriptomic studies have shown the presence of T_RM_-like cells in human tissues and tumors, and the density of these cells within human tumors correlates with improved prognosis^17,18,64^. In our study, we demonstrate that IRE in combination with anti-CTLA-4 boosts endogenous T_RM_ formation in numerous NLT, and is associated with protection from recurrent tumor challenge. By contrast, we noted that despite similar proportions of circulating tumor-specific memory CD8+ T cells at later time points, mice that received monotherapy anti-CTLA-4 failed to control a recurrent tumor challenge. Although we cannot extricate the contribution of circulating tumor-specific CD8+ T cells in secondary tumor control, these results suggest endogenous tumor-specific T_RM_ at distal sites can aid in tumor protection. Previous studies have demonstrated that self-specific T cells can be mobilized to control tumor and form T_RM_ populations following heterologous viral boosting, particularly in combination with ICI therapy^65,66^. Our study further extends these observations by describing the differentiation and establishment of neoantigen-specific CD8+ T_RM_ in diverse NLTs from the endogenous T cell pool. We also confirm previous work showing that there is little overall impact of ICI and other immunomodulatory monotherapy on tumor growth and protection in this model^35,58,67^, echoing the clinical need for expanded options in combinatorial therapy.

Overall clinical objective response rates to ICI remain limited to a relatively small subset of patients, even for the most immunogenic tumors. For poorly immunogenic cancers, there remains an acute need for neoantigen release and concomitant T cell priming and expansion. In pancreatic cancer, T cell repertoire diversity and vaccines that enrich dendritic cells are associated with modest positive responses to anti-PD-1/anti-PD-L1 ICI^68–70^. Similarly, in the most aggressive subsets of castration-resistant prostate cancer (CRPC), the dendritic cell vaccine Sipuleucel-T is the only approved immunotherapy for CRPC but of modest benefit^71^. Anti-CTLA-4 and anti-PD-1 therapy of T-cell infiltrated subsets of CRPC is associated with T cell expansion but compensatory immunosuppression^72^, with best outcomes achieved only when considering those patient cohorts with the highest neoantigen favorability scores^73^. The TRAMP-C2 cells used in our study remain dependent on androgens for growth, which is a limiting factor. However, it should be noted that complex pathways regulate androgen signaling and androgen hypersensitivity, and that most clinical CRPC retains androgen dependence as evidenced by the efficacy of the androgen synthesis inhibitor abiraterone acetate for treatment of CRPC^74–76^. Therefore, our work with the androgen-dependent TRAMP-C2 model is likely to have relevance to CRPC, and indicates that many CRPC tumors may benefit from novel combinations of anti-androgen and immune checkpoint therapy.

In our study, application of IRE prior to anti-CTLA-4 therapy promoted profound changes in the neoantigen-specific T cell response, resulting in increased numbers of SPAS-1+ T cells systemically in the spleen and concentrated in the tumor and distant sites. Anti-CTLA-4 appeared to increase the priming of T cells, as bulk CD8+ T cells were increased following anti-CTLA-4 treatment, and bulk intratumoral CD8+ T cells maintained IFN-gamma production. However, the number of intratumoral regulatory (CD4 + FoxP3+) T cells was not affected when probed following therapy. Thus, while the precise mechanism of action of anti-CTLA-4 is unclear in our model, it does not appear to involve chronic depletion of regulatory T cells. The SPAS-1+ tumor-specific T cells maintained evidence of functionality as evidenced by their exclusion from the tumor vasculature, access to the tumor tissue, and maintenance of cytokine secretion capacity. Further, the response to dual therapy was in contrast to that observed following IRE monotherapy, in which non-expanded SPAS-1+ cells were typified by increased expression of exhaustion markers including PD-1, LAG-3, and the exhaustion-associated transcription factor TOX. Our combinatorial data are consistent with the hypothesis that the application of tumor ablative technology prior to the introduction of ICI is a rational mechanistic strategy to invoke strong tumor-specific immune responses. This is particularly significant in light of the prediction that therapies that generate CD8+ T_RM_ subsets will be associated with durable immunosurveillance^19,20^.

Kinetic tracking of the neoantigen-specific CD8+ T cell response revealed key aspects of the response timing. Our analysis included identifying the naive precursors and tracking their initial activation (Fig. 1) and distribution changes in blood, tumor, and tissues after tumor challenge (Figs. 2–3) and ICI therapy (Figs. 5–7). We found that anti-PD-1 as a primary combination following IRE treatment did not affect SPAS-1+ CD8+ T cell expansion or tumor outcome in our study. By contrast when we administered anti-PD-1 later, at the peak of SPAS-1+ T cell expansion invoked by IRE plus anti-CTLA-4, we observed sustained control of tumor growth, particularly in those mice that did not respond fully to initial dual therapy (Fig. 9). Despite high expression of PD-1 on SPAS-1+ T cells following IRE monotherapy (Fig. 6h), we attribute the lack of effect of anti-PD-1 immediately following IRE treatment to impaired T cell priming and proliferation, consistent with the observation that circulating levels of SPAS-1+ CD8+ T cells were not increased when IRE was followed by anti-PD-1 treatment. Together, these data suggest that the proposed actions of anti-CTLA-4 on extending T cell priming following antigen release are critical and distinct from anti-PD-1, which likely targets expanded but partially exhausted T cells in tumors^35,77^. Thus, mechanistic windows for temporal application of ICI therapy appear to exist in our system, although future studies in additional translatable models will be required to extend this observation. Besides validating an intervention scheme for combinatorial use of two distinct ICI drugs, our work provides a rational basis for consecutive application of ICI agents rather than parallel administration, which is consistently associated with severe immunopathology and loss of patient compliance^78^.

A wide range of tumor treatment techniques, including radiation therapy, chemotherapy, and focal therapy have been studied for invoking anti-tumor immune responses, especially for improving local and systemic cancer control in combination with ICIs^28,32,79–81^. Recently focal therapy has emerged as a viable alternative treatment option for small, localized prostate tumors^32,40^. With advances in probe design and in vivo imaging guidance, IRE is an emerging technique that couples increased antigen preservation with precise destruction in a complex tumor structure, and mitigates the heat sink effect created by blood flow during thermal and cryosurgical methods^39,40^. The mode of cell death and extent of local inflammation likely play a central role in in situ vaccination for anti-tumor immunity. The mechanism of action induced by different types of focal tumor ablation remains under-investigated but presumably involves dendritic cell cross-presentation of released tumor neoantigens to T cells^27,82,83^. Our observation that IRE monotherapy is associated with dysfunctional tumor-specific T cells in tumors and regional tumor-draining lymph nodes, and partial exclusion from tumors and/or trapping in the tumor vasculature, may provide important mechanistic guidance for clinicians evaluating the impact of local focal ablation. It is possible that IRE-induced modulation of the tumor stroma, extracellular matrix, and/or vasculature may account for some of these changes. This may provide insight into more strategic timing and location of biopsy collection, and determining optimal time windows for the administration of anti-CTLA-4 or anti-PD-1 ICI without over-prescribing these agents. This is particularly relevant given recent reports that increased tumor-infiltrating and systemic T cell responses have been observed after IRE treatment in vivo^45,46,52,53^. Clinically, a transient decrease in systemic regulatory T cells and a boost in tumor antigen-specific T cell responses have also been observed in patients with locally advanced pancreatic cancer treated with IRE^42,48^. However, the significance and dynamics of these types of changes on tumor neoantigen-specific T cell expansion following IRE remain unclear.

In addition to the immunomodulatory effect of focal tumor ablation alone, these techniques are being combinatorially leveraged to increase the efficacy of immunotherapy. Recent studies of IRE in murine pancreatic tumor models have demonstrated increased levels of MHC-II and inflammatory and costimulatory antigen presentation protein levels on dendritic cells, consistent with a role in antigen presentation^82^. These combinatorial therapy studies indicate changes in the local tumor and regional lymphatic microenvironment during tumor therapy^52,53^ that will require further investigation. In addition, previous studies have emphasized the need to monitor abscopal effects and protection from secondary tumor challenge to test efficacy of tumor-specific immunity following focal tumor ablation and the administration of ICI^35,53^. Our study specifically tracked endogenous tumor neoantigen-specific T cells from the point of induction into their relevant site of action in tumor-distal NLTs, following combined tumor ablation and ICI therapy. This work confirmed that distant tumor-specific resident memory T cells can be augmented by efficacious combination therapy, and are consistent with the possibility of intratumoral tissue residency, but additional studies will be required to determine the appropriate phenotypic markers and design of migration experiments to define the formal residency status of endogenous tumor-neoantigen-specific memory T cells.

Future studies investigating the nature of CD8+ T cell subsets in responses to combinatorial focal therapy and immunotherapy are warranted, including identifying treatment conditions and interventions that can further influence tumor antigen-specific CD8+ T cell programming and tissue trafficking properties. Our work is consistent with the model that boosting endogenous tumor-specific T cells using combination therapy can generate distant, whole-body distribution of protective tissue-resident memory cells. Use of this information with emphasis on the concerted impact of thermal and non-thermal tumor ablation on antigen preservation and local inflammation will inform refinement of these technologies. Furthermore, assessment of the biodistribution and function of tumor-specific immune responses between the tumor, circulation, and peripheral tissues^21^ will be important for improving the translational and clinical application of ICI in pursuit of combined therapies that function as an effective in situ vaccination.

## Methods

### Mice

All procedures performed on mice were approved by the Institutional Animal Care and Use Committee (IACUC) at the University of Minnesota and conformed to all relevant ethical regulations for animal testing and research. C57BL/6J mice were obtained from Jackson Labs at age 8–10 weeks unless noted. Male mice were used for TRAMP-C2 tumor inoculation, unless indicated. Mice were housed in a specific pathogen-free (SPF) vivarium at the University of Minnesota. Housing conditions consisted of a 14/10 h light/dark cycle at 20.0–23.3 C and 30–70% saturating humidity.

### Cell lines

Transgenic adenocarcinoma of the mouse prostate-C2 (TRAMP-C2) cells were obtained from the American Type Tissue Collection (ATCC) as mycoplasma-free stocks designated as passage 0, and cultured on tissue culture plastic (Corning Life Sciences) in Dulbecco’s Modified Eagles Medium with 4.5 g/L glucose (Gibco) supplemented with 5% fetal bovine serum (FBS, Gibco), 5% Nu-Serum (Corning 355100), 100 U/mL penicillin, 100 µg /mL streptomycin, 4mM L-glutamine, 10 mM HEPES, 5 µM 2-mercaptoethanol (Gibco), 5 µg/mL insulin (Sigma I9528), 10 nmol/L dihydrotestosterone (Sigma DO77). TRAMP-C2 cells were lifted for passage using trypsin or Tryp-LE (Gibco) followed by washing with complete culture medium, and frozen into working stock vials at passage 2. Working vials were thawed and passaged <2–3 additional times and finally passaged ~1:2 the day prior to tumor implantation. Subcutaneous (s.c.) inoculation was performed by injecting 0.4 × 10^6^ cells in a 200 µl volume of PBS under the hindlimb (flank) skin, using a 27 1/2 gauge insulin syringe. Intradermal (i.d.) injection of TRAMP-C2 cells in matrigel was performed for mice in experiments where IRE was used, and for secondary tumor challenge in cured mice, to allow spheroid tumors to form. This injection was performed under brief isoflurane anesthesia on a shaved ~1 cm^2^ region of the flank. Matrigel (EHS tumor matrix, Corning) was thawed on ice and diluted 1:1 with TRAMP-C2 cells in ice-cold PBS to achieve a final dose of 1 × 10^6^ cells per 50 µl injection into the dermis using a chilled 29 ½ gauge syringe. Tumors were measured every 2–3 days using a caliper. Tumor size was calculated by measuring the longest and shortest dimensions, and multiplying to provide a two- dimensional representation of the tumor area^16,84^.

### Immunotherapy

TriVax vaccinations were performed as previously described^57^, using 50 µg peptide, 100 µg anti-CD40 (Clone FGK4.5), and 50 µg Poly (I:C)(InvivoGen). Poly (I:C) was heated to 65–70 °C for 10 min, cooled to room temperature, and stored at −20 C prior to assembling vaccination doses in 200 µL for intravenous injection.

Stock solutions of anti-CTLA-4 (clone 9H10), anti-PD-1 (clone RMP1-14), anti-PD-L1 (clone 10 F.9G2), and control immunoglobulin (IgG) were purchased from Bio X Cell. Antibodies were diluted in sterile PBS to 1 mg/mL working concentrations, and unless otherwise noted were injected into the peritoneal cavity at 200 µg followed by 100 µg every third day, for 4 total injections.

### Surgery

Two hours prior to the following procedures, mice were pre-treated with subcutaneous buprenorphine SR (2 mg/kg) analgesic, and then sedated for the procedure by intraperitoneal administration of ketamine (100 mg/kg) and xylazine (10 mg/kg).

IRE was performed using a custom probe consisting of a sliding pair of stainless steel parallel plate electrodes. Briefly, 5–6 mm diameter tumors with a ~1:1 aspect ratio were enrolled for IRE treatment. Hair was removed from the tumor and surrounding area using a mechanical razor followed by nair treatment, and the skin washed with water and dried using a kimwipe. The tumor was pinched by the IRE probe, with the distance measured by a caliper. The probe was connected to a pulse generator (ECM 830 electroporator, BTX) to provide an electric field of 1500 Volts/cm through the tumor. Two sets of 25 monophasic direct current pulses (1 Hz, 50 µs pulse width) were applied in two orthogonal directions to ensure complete coverage of the tumor by the electric field. Sham-treated control tumors were prepared and identically pinched with the electrode as with IRE, but no voltage applied.

Surgical resection was performed on 5–6 mm diameter tumors as previously described^85^. Briefly, tumor-bearing mice were pretreated and anesthetized as described above. The hair above the tumor area was cleared by shaving and nair treatment, and sterilized using Betadine solution and 70% isopropyl alcohol pads. A small incision adjacent to the tumor was made using sterile surgical scissors and the intradermal tumor mass excised, including a ~2 mm excess margin. Surgical glue (3 M Vetbond) was used to attach the residual opposing skin flaps, which were also anchored using three surgical wound clips. Mice were recovered on heating pads until fully awake before being returned to home cages.

Parabiotic surgery was performed under analgesia and anesthesia as described above, using previously described methods^13,86^. Briefly, mice were shaved on opposite flanks and opposing skin was cleared of hair as described above. The site was sterilized using Betadine solution and 70% isopropyl alcohol pads. Lateral mirrored incisions were made on both mice. The lateral skin incisions on both mouse pairs were then conjoined with surgical wound clips. Equilibration between both partners was confirmed in the peripheral blood 14 days after surgery. Parabionts were allowed to rest for 17 days before harvest and tissue analysis.

### Tissue processing

Mice were euthanized by cervical dislocation under isoflurane anesthesia. In some experiments, 200 µl of PBS containing 3 µg of FITC or PE-Cy7 conjugated anti-CD8α antibody (clone 53-6.7, Tonbo Biosciences) was introduced into the bloodstream by retro-orbital injection 3 min prior to euthanasia, to mark circulating CD8+ T cells^11^. Single-cell suspensions were generated as previously described^86–88^. Briefly, organs were collected in and all rinses performed using harvest buffer (HB) consisting of RPMI-1640 (Corning) containing 5% FBS, 10 mM HEPES and 4mM L-glutamine. Spleens and lymph nodes were dissociated by scraping over an etched plastic dish with a flat syringe plunger. The liver was pushed through a 70 µm strainer using a flat plunger, followed by isolation of lymphocytes from the interface of a 44%/66% Percoll step gradient^11^. Blood, spleen, and liver samples were treated with hypotonic saline (ACK buffer) to lyse red blood cells. All other tissues were transferred into GentleMacs tubes (Miltenyi) containing 0.5 mL of HB and rapidly minced into ~1 mm pieces using microdissection scissors. Digest Buffer (HB containing 1 mM each of CaCl_2_ and MgCl_2_, and 1 µg/mL DNAse I (Sigma DN25) was supplemented with collagenase as follows and was added to each sample to 10 mL final volume, followed by incubation at 37 °C in an angled rack with shaking at 250 rpm. Salivary glands and lungs were digested for 45 and 60 min, respectively, in 200 U/mL of collagenase I (Worthington CLS-1). The kidney and female reproductive tract were digested for 30 mins and 60 min, respectively, in 0.5 µg/mL of collagenase IV (Sigma C5138). Tumors were digested in 100 U/mL of collagenase I and 0.5 mg/mL collagenase IV. Hair was shaved from a flank skin sample (distal from the tumor, if present) and fat removed by scraping with a forcep handle. A 2 cm^2^ of skin was minced then added to 5 mL of digest buffer containing 2 mg/mL collagenase IV and 1 µg/mL DNAse for 60 min at 37 °C with shaking. Enzymatically digested tissues were further dissociated by running the Spleen.01 program on a GentleMacs dissociator (Miltenyi). All samples were then passed through 60 µm nylon mesh and resulting single-cell suspensions were centrifuged and resuspended in a defined volume of FACS buffer (HBSS containing 0.1% sodium azide and 2% bovine serum), prior to being transferred to 96-well plates for staining with fluorescent antibody cocktails and MHC-I tetramers.

### MHC-I tetramers and tetramer enrichment

Major Histocompatibility Class I (MHC-I) restricted tetramers loaded with the SPAS-1 peptide “H8” STHVNHLHC (New England Peptide)^35,36^ were produced as monomer and stored and tetramerized with streptavidin-PE and –APC (Invitrogen) as previously described^89^.

Magnetic enrichment and staining using MHC-I tetramers were performed as previously described^56,90^. Briefly, spleens and lymph nodes (inguinal, brachial, axilliary, cervical, and mesenteric) from naive and tumor-challenged mice were collected in HB and dissociated in a Gentle Macs tube on setting Spleen.01 (Miltenyi Biotech). Samples were filtered and resuspended into a 200 µl volume of FACS buffer containing 1% rat serum and 0.5 µg/mL Fc-block, and incubated with 2 µg each of PE- and APC- conjugated SPAS-1 tetramer for 60 min at RT. Samples were washed and incubated for 15 min at RT with anti-APC and anti-PE beads as directed by the manufacturer (Miltenyi Biotech), and washed again prior to capture on magnetic separation columns. Columns were washed three times to remove unbound cells and the bound fraction removed from the magnet and collected and processed for flow cytometry.

### Flow cytometry

Aliquots of single-cell suspensions or tetramer-enriched fractions were stained for flow cytometry in a 50 µl volume of Tonbo staining buffer (Tonbo Biosciences) containing a cocktail of fluorescently conjugated antibodies. Final staining dilution was 1:100 for all antibodies, unless otherwise indicated. Ghost Dye Red 780 viability stain (1:200), APC-Cy7 conjugated anti-mouse/human CD45R/B220 (clone RA3-6B2), and APC-Cy7 conjugated anti-mouse MHC-II (I-A/I-E)(clones M5/11 4.15.2) used for exclusion gating were from Tonbo Biosciences. FITC and BV785 conjugated anti-mouse/human CD44 (Clone IM7) were from Tonbo Biosciences and Biolegend, respectively. FITC and PE-Cy7 conjugated anti-mouse CD8α (clone 53-6.7) was from Tonbo Biosciences and BV421 and BV785 conjugated anti-mouse CD8α (clone 53-6.7) was from Biolegend. BV421 conjugated anti-mouse CD69 (1:50), and BV605 conjugated anti-mouse CD62L (clone MEL-14) and PerCP-Cy5.5, PE-Cy7, and BV785 conjugated anti-mouse PD-1 (clone 29F.1A12) were from Biolegend. BV421 and BV711 conjugated TIM-3 (clone RMT3-23) and LAG-3 (clone C9B7W) and BV510 conjugated CD103 (clone 2E7) were from Biolegend. FITC anti-mouse CD45.2 (clone 104) was from Tonbo. Thy1.2 AF700 (1:300, clone 30-H12) was from Biolegend. BUV395 anti-mouse Thy1.2 (Clone 53-2.1)(1:300), BUV395 anti-mouse CD45.1 (Clone A20)(1:50), BUV495 anti-mouse CD4 (Clone GK1.5), BUV737 anti-mouse CD8a (Clone 53-6.7) and BV711 anti-CD49a (clone Ha31/8) were from BD Biosciences. For intracellular staining, anti-human/mouse TOX eFluor 660 (clone TXRX10) was from ThermoFisher, PerCP-Cy5.5 anti-IFN-gamma (clone XMG1.2) was from Tonbo and BV421 anti-mouse TNFα (clone MP6-XT22) was from Biolegend.

Samples were stained in FACS tubes or 96-well plates for 30 min at 4 °C and washed one or two times, respectively, with excess ice-cold FACS buffer (HBSS containing 2% bovine serum). For samples not pre-enriched with tetramer, staining was performed for 60 min at room temperature. For restimulation, single-cell suspensions representing 1/10th of the spleen or 1/5th of tumor samples were incubated in 200 μl of HB containing 10% FBS and 1× cell stimulation and protein transport inhibitor cocktails (eBioscience) for 3.5 h at 37 C, prior to cell surface staining. Intracellular staining for all targets was performed using the FoxP3/transcription factor kit according to the manufacturer protocol, with overnight intracellular antibody incubation at 4 C. Following staining, samples were washed and resuspended in PBS containing 0.5% paraformaldehyde, and a known number of PKH Counting Beads (Sigma) was added to permit quantification of absolute cell numbers within flow cytometric frequency gates. Flow Cytometry was performed on BD LSRII or Fortessa X-20 or X-30 cytometers in the University of Minnesota Flow Cytometry Core Facility (UFCR) using FACS Diva software version 8.0.1, and analyzed using FlowJo software (Treestar, Inc) version 10. Tissue residency in individual parabionts was calculated in each tissue by dividing the absolute number of gated SPAS-1+ CD8+ T cells derived from the host, by the total number of SPAS-1+ CD8+ T cells from both the host and the partner for that tissue.

### Statistics and reproducibility

Graph Pad Prism software version 9 was used to perform statistical analysis. All data points refer to and constitute biologically independent (replicate) mice. D’Agostino and Pearson’s test was used to determine whether data fit Gaussian distributions. When data followed a normal distribution, we used parametric tests (unpaired, two-tailed Student’s *T*-test or one-way ANOVA). When ANOVA *p* < 0.05, we used Tukey’s multiple comparison test to compare more than two groups, or Dunn’s multiple comparison test when comparing multiple groups to a single group. For non-normally distributed data, non-parametric tests were used (Mann–Whitney *U* test or Kruskal–Wallace). Unless indicated, ordinary one-way ANOVA was used to compare more than two groups. For comparison of two groups, unpaired two-tailed Mann–Whitney or Student’s *T*-tests were used unless otherwise indicated. The significance of Kaplan–Meier curves was determined by Mantel-Cox log rank tests. Where possible, exact two-sided *P* values are displayed on figure panels. In all cases unless indicated, *P* values are: *p < 0.05; **p < 0.01; ***p < 0.001; ****p < 0.0001. Unless otherwise indicated, all error bars reflect the mean ± the standard error of the mean (S.E.M.).

### Reporting summary

Further information on research design is available in the Nature Research Reporting Summary linked to this article.

## Supplementary information

Burbach et al Supplementary Information

Reporting Summary

## Data Availability

Source data are available as a Source Data File. The remaining data are available within the Article and Supplementary Information. Source data are provided with this paper.

## Source data

Source Data File
