# Supplementary material for: Irreversible electroporation augments checkpoint immunotherapy in prostate cancer and promotes tumor antigen-specific tissue-resident memory CD8+ T cells
Source: Nat Commun. 2021 Jun 23;12:3862. doi: 10.1038/s41467-021-24132-6 (PMC8222297; doi:10.1038/s41467-021-24132-6)
Supplement: Supplementary file 1 — Burbach et al Supplementary Information [file 41467_2021_24132_MOESM1_ESM.pdf]

SUPPLEMENTARY INFORMATION FILE

Irreversible electroporation augments checkpoint immunotherapy in prostate cancer and promotes tumor antigen-specific tissue-resident memory CD8$^+$ T cells

Brandon J. Burbach[1,2,3,4,12,*], Stephen D. O'Flanagan[1,2,5,12], Qi Shao[3,4,6,12], Katharine M. Young[1,2], Joseph R. Slaughter[1,2], Meagan R. Rollins[1,2,7], Tami Jo L. Street[1,2], Victoria E. Granger[1,2], Lalit. K. Beura[2,5,11], Samira M. Azarin[3,4,8], Satish Ramadhyani[9], Bruce R. Forsyth[7], John C. Bischof[3,4,6,10], and Yoji Shimizu[1,2,3,4,*]

[1]Department of Laboratory Medicine and Pathology, University of Minnesota, Minneapolis, MN
[2]Center for Immunology, University of Minnesota
[3]Masonic Cancer Center, University of Minnesota
[4]Institute for Engineering in Medicine, University of Minnesota
[5]Department of Microbiology and Immunology, University of Minnesota
[6]Department of Mechanical Engineering, University of Minnesota
[7]Boston Scientific Corporation, Maple Grove, MN
[8]Department of Chemical Engineering and Materials Science, University of Minnesota
[9]BTG plc, Arden Hills, MN
[10]Department of Biomedical Engineering, University of Minnesota
[11]Present Address, Department of Molecular Microbiology and Immunology, Brown University, Providence, RI
[12]These authors contributed equally to this work

* Address correspondence to:

Brandon J. Burbach, PhD
Yoji Shimizu, PhD
University of Minnesota, Center for Immunology
2-200 Medical Biosciences Building
2101 6th St SE, Minneapolis, MN 55455
Email: burba005@umn.edu
Email: shimi002@umn.edu
Tel: 612-626-6713 (BJB)
Tel: 612-626-6849 (YS)
Fax: 612-625-2199

Running Title: IRE augments immunotherapy and promotes CD8+ T_{RM}

Supplementary Figure 1

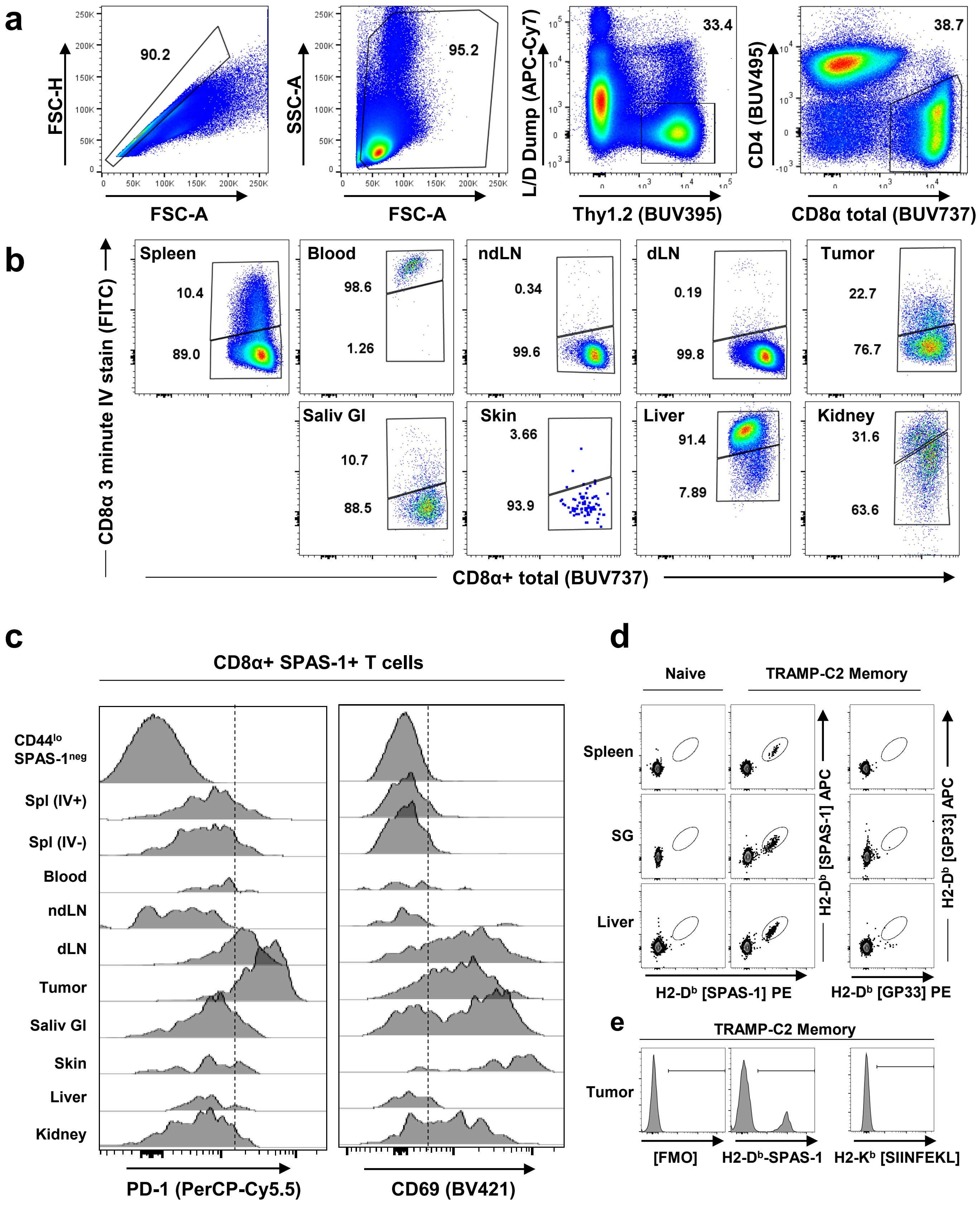

Supplementary Figure 1. Phenotype of SPAS-1+ T cells distributed in lymphoid and non-lymphoid tissues following TRAMP-C2 tumor challenge. See also **Figure 2**. Mice were challenged s.c. with TRAMP-C2 in the flank necropsy performed 28 days later. Mice were individually injected i.v. with 3 µg of FITC-labelled anti-CD8α antibody 3 min prior to harvest, to mark CD8+ T cells in the circulation. **a** Representative flow cytometry gating of stained single cell suspensions (shown for spleen). Dump indicates staining with B220, MHC-II, and live/dead viability dye in a single channel for non T cell lineage exclusion gating. Identical or very similar gating used for all figures. **b** Representative gating of total CD8+ T cells used to discriminate vascular (IV+) and non-vascular (IV-) CD8+ cells. **c** Representative histograms showing PD-1 (left) and CD69 (right) staining of SPAS-1+ T cells from the indicated tissues. Abbreviations: IV, intravascular (- or +); Spl, Spleen; dLN, tumor-draining lymph node; ndLN, non-draining LN. **d** Flow cytometry plots showing example control staining of single cell suspensions of the indicated tissues from naïve or day 35 TRAMP-C2 tumor challenged mice, stained with H2-D^b [SPAS-1] as in Figure 2. Right column, staining with H2-D^b tetramers loaded with a non-cognate GP33 peptide as a control. **e** Flow cytometry plots showing example control staining of single cell suspensions from TRAMP-C2 tumor. FMO, fluorescence minus one control omitting H2-D^b [SPAS-1] PE labelled tetramer. Right panel, H2-K^b [SIINFEKL] PE was used as an additional non-cognate tetramer control.

Supplementary Figure 2

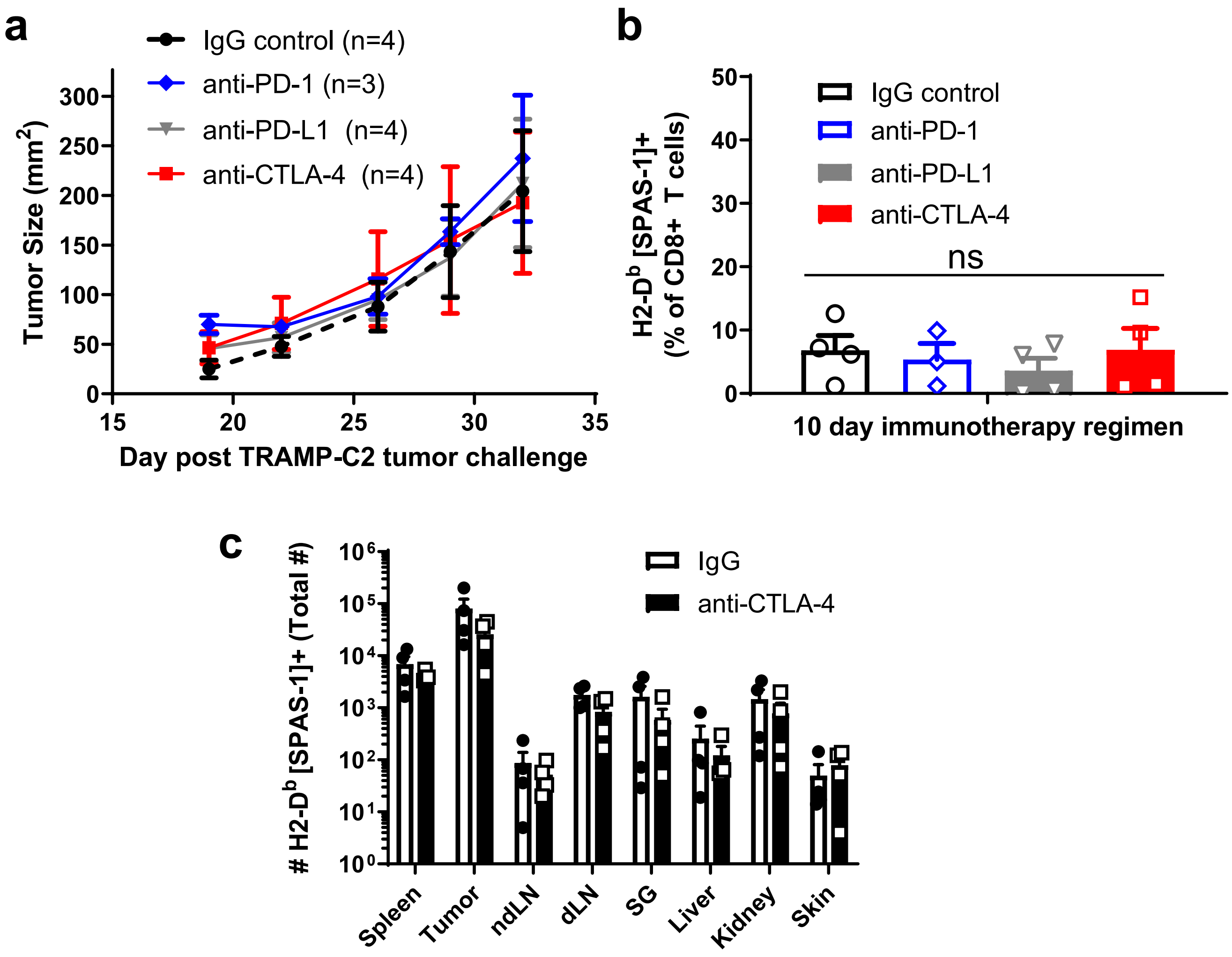

Supplementary Figure 2. TRAMP-C2 tumor growth and SPAS-1 T cell density and distribution is unaffected by ICI immunotherapy. C57BL/6J mice were challenged s.c. with TRAMP-C2 tumor cells. On day 18 when tumors were approximately 4-5mm diameter, mice were treated IP with 200 µg of the indicated antibody therapy, followed by 100 µg injections on day 21, 24, and 27. **a** Measurement of tumor growth over time, n=3-4 animals per group, with exact n shown for each group. Results are representative of two independent experiments performed. **b** Flow cytometry was used on Day 33 to determine the frequency of SPAS-1+ CD8+ T cells in the tumors of each mouse in **a**. Kruskal-Wallace test was performed; ns, not significant. Results are representative of two experiments performed. **c** Mice bearing TRAMP-C2 tumors were treated with IgG or anti-CTLA-4 as in **a**. At day 35, mice were euthanized 3 minutes following intravascular labelling of circulating CD8+ T cells. The absolute number of SPAS-1+ CD8+ T cells in each tissue (excluded from vasculature) was determined by flow cytometry as in Figure 2. Data points represent biologically independent mice (n=4/group) from one experiment representative of two similar experiments performed. Abbreviations: SG, salivary gland; ndLN, non-draining lymph node; dLN, tumor-draining lymph node. Bars represent mean ± S.E.M. Source data are provided as a Source Data File.

Supplementary Figure 3

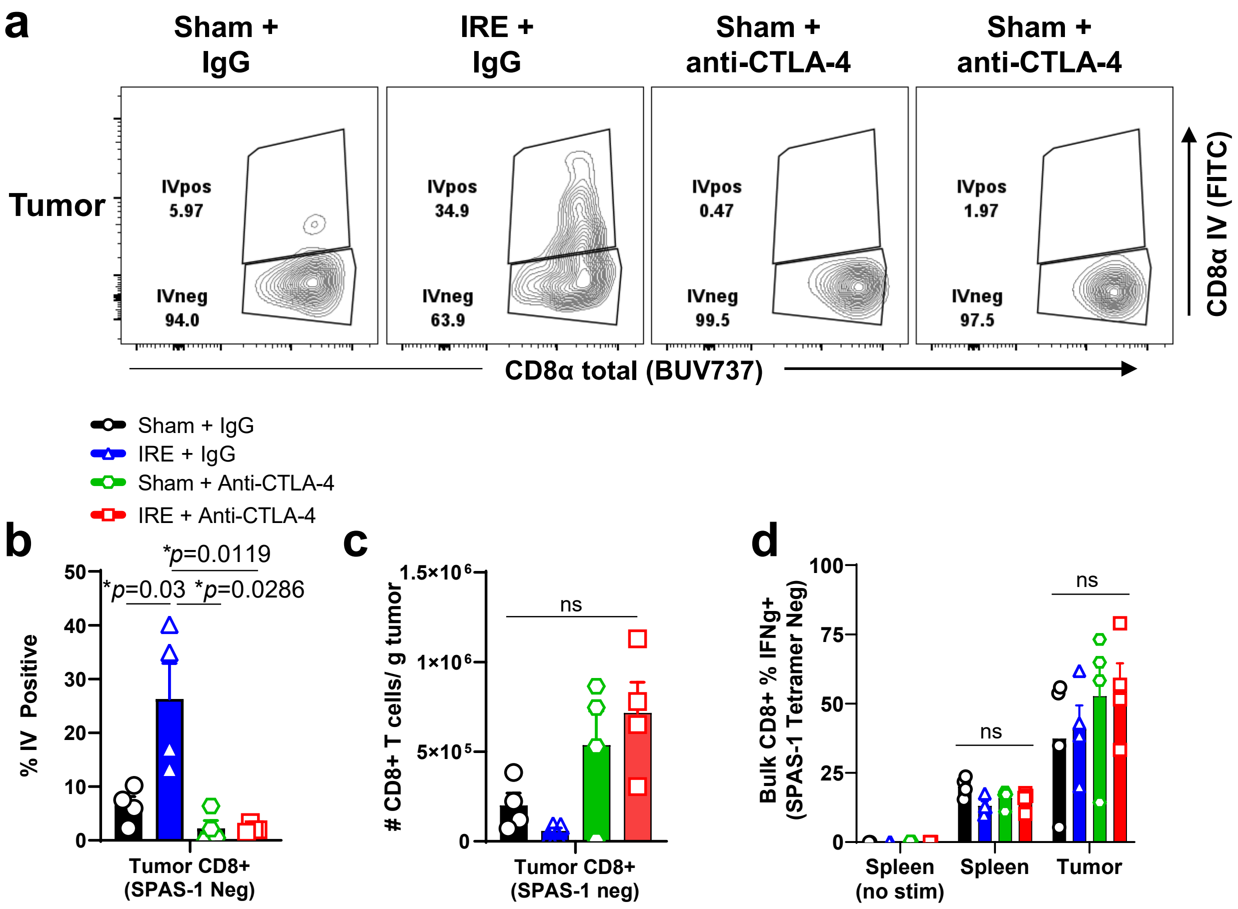

Supplementary Figure 3. Analysis of tetramer-negative bulk CD8+ T cells following combination therapy. C57BL/6J mice were challenged s.c. with TRAMP-C2 tumor cells and mice bearing ~5mm diameter tumors were treated with Sham, IRE, and/or anti-CTLA-4 as described in Figure 6. Single cell suspensions from the indicated tissues were analyzed by flow cytometry on day 14 following therapy onset. Intravascular (IV) anti-CD8α was injected 3 minutes prior to sacrifice. **a** Representative flow cytometry plots gated on Thy1.2+ CD8+ SPAS-1 negative (bulk CD8α+ tetramer-negative) T cells, showing IV CD8α stain used to segregate the IV-negative and -positive fractions from each treatment group. **b-c** Quantification of bulk CD8+ IV staining and enumeration of total CD8+ tetramer-negative T cells, respectively. **d** Intracellular staining for cytokine production following restimulation. Data points in **b-d** represent all individual mice (n=4/group) pooled from two independent experiments. Bars represent mean ± S.E.M. Unpaired two-tailed Mann-Whitney Test was performed; *, p <0.05; ns, not significant. Source data are provided as a Source Data File.

Supplementary Figure 4

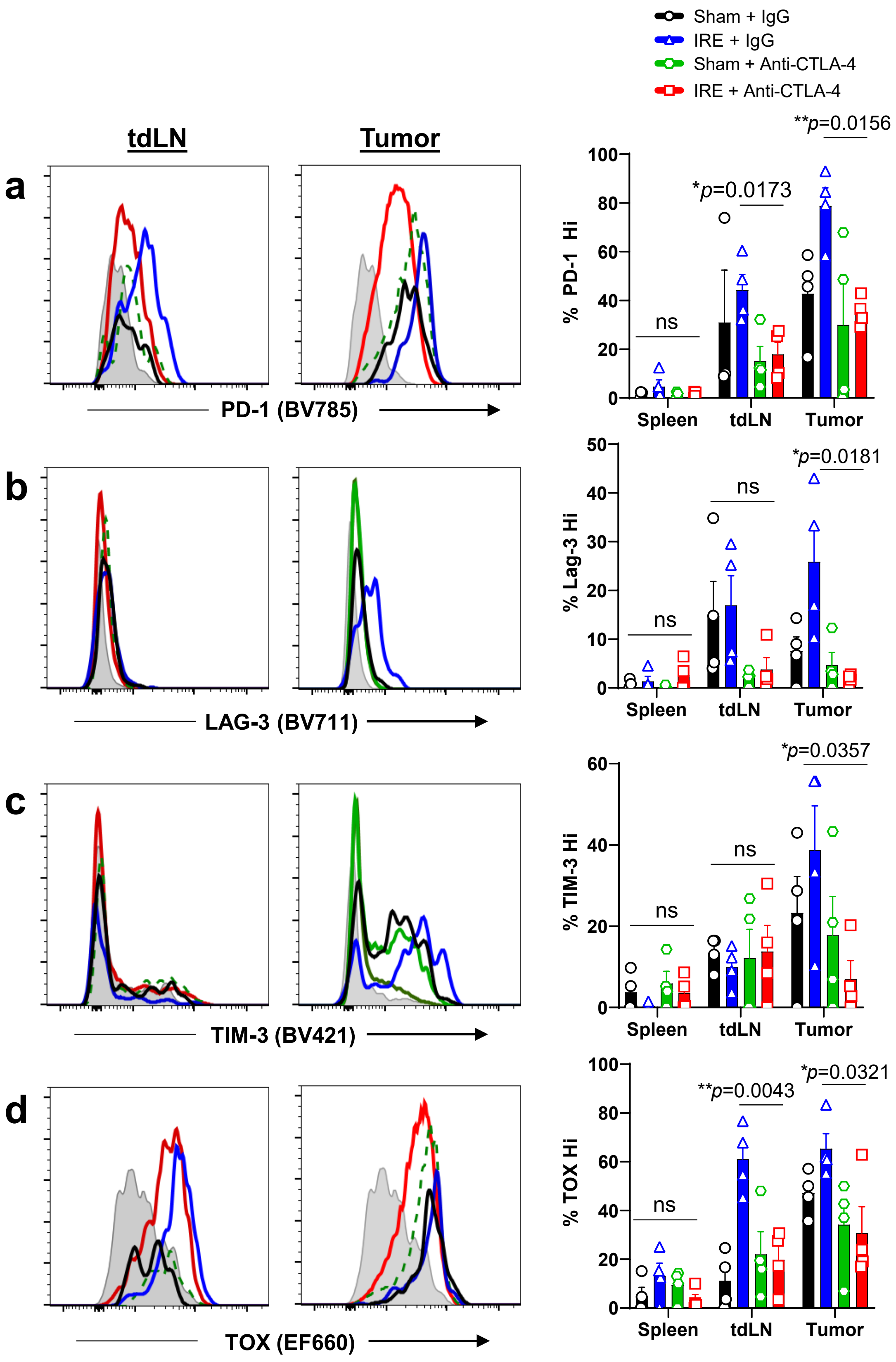

Supplementary Figure 4. Expression of inhibitory molecules on SPAS-1+ T cells following combination therapy. C57BL/6J mice were challenged s.c. with TRAMP-C2 tumor cells and mice bearing ~5mm diameter tumors were treated with Sham, IRE, and/or anti-CTLA-4 as described in Figure 6. Single cell suspensions from the indicated tissues were analyzed by flow cytometry on day 14 following therapy onset. Thy1.2+ CD8+ SPAS-1+ T cells from the IV-negative gate are shown. **a-d** Histogram overlays from representative tumor samples from each condition, with quantification to the right of each overlay showing data points representing all individual mice (n=4/group) pooled from two independent experiments. Gray control histogram represents SPAS-1+ cells from the spleen IRE + anti-CTLA-4 condition. **a-c** Cell surface PD-1, LAG-3, and TIM-3 expression, respectively. **d** Intracellular staining for TOX expression. Abbreviation: tdLN, tumor-draining lymph node. Bars represent mean ± S.E.M. Unpaired two-tailed Mann-Whitney test was performed; *, $p<0.05$; **, $p<0.01$ ns, not significant. Abbreviation: tdLN, tumor-draining lymph node. Source data are provided as a Source Data File.

Supplementary Figure 5

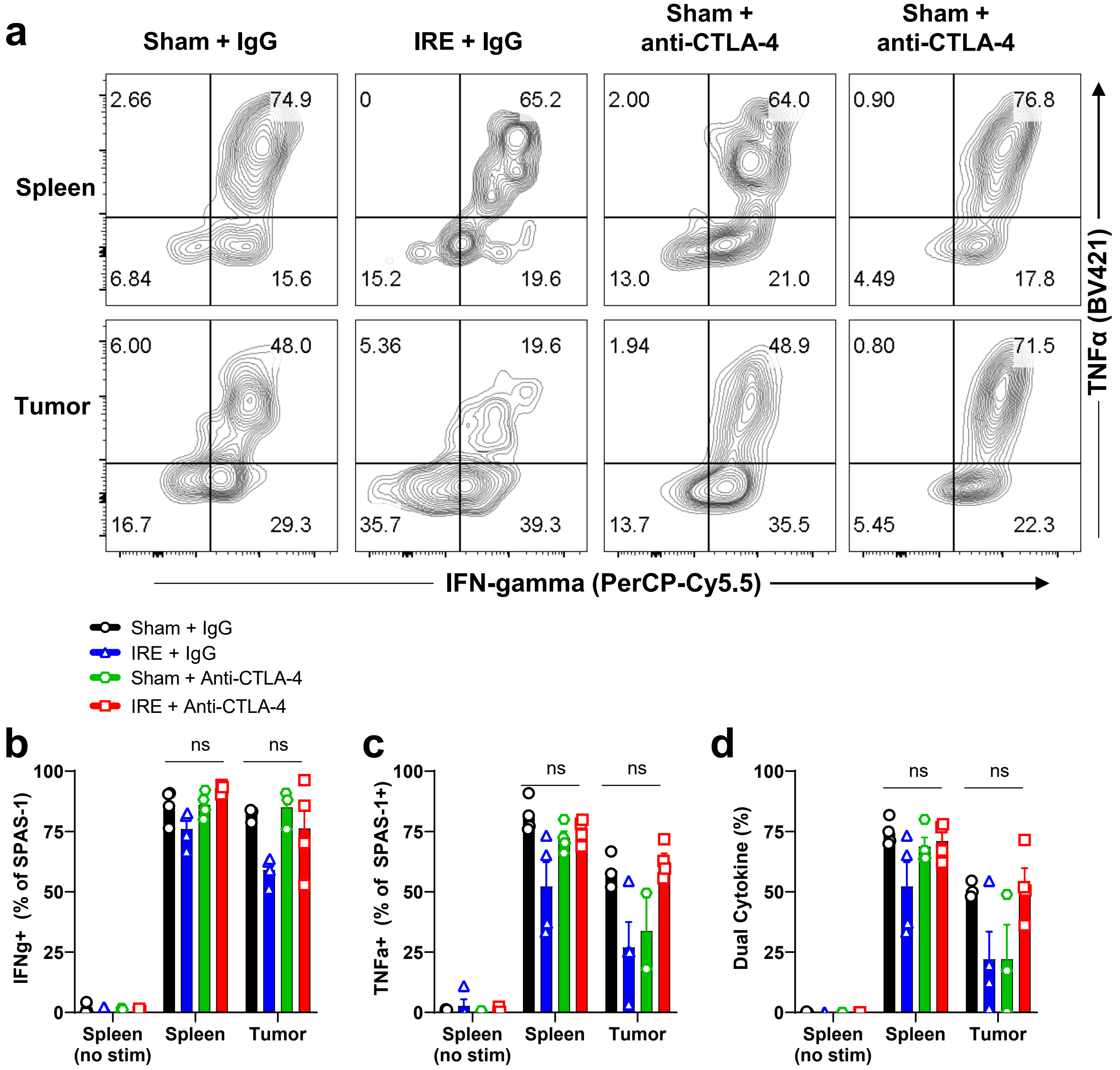

Supplementary Figure 5. Cytokine production by SPAS-1+ T cells following combination therapy. C57BL/6J mice were challenged s.c. with TRAMP-C2 tumor cells and mice bearing ~5mm diameter tumors were treated with Sham, IRE, and/or anti-CTLA-4 as described in Figure 6. Single cell suspensions from the indicated tissues were analyzed by flow cytometry on day 14 following therapy onset. Samples were restimulated for 3.5 hours prior to sequential surface and intracellular staining. Thy1.2+ CD8+ SPAS-1+ T cells from the IV-negative gate are shown. **a** Representative flow cytometry plots showing cytokine production, with gates set against unstimulated control samples. **b-d** Quantification of intracellular cytokine staining for IFN-gamma, TNFα, and dual cytokine production, respectively. Data points in **b-d** represent all individual mice (n=4/group) pooled from two independent experiments. Bars represent mean ± S.E.M. ns, not significant. Source data are provided as a Source Data File.

Supplementary Figure 6

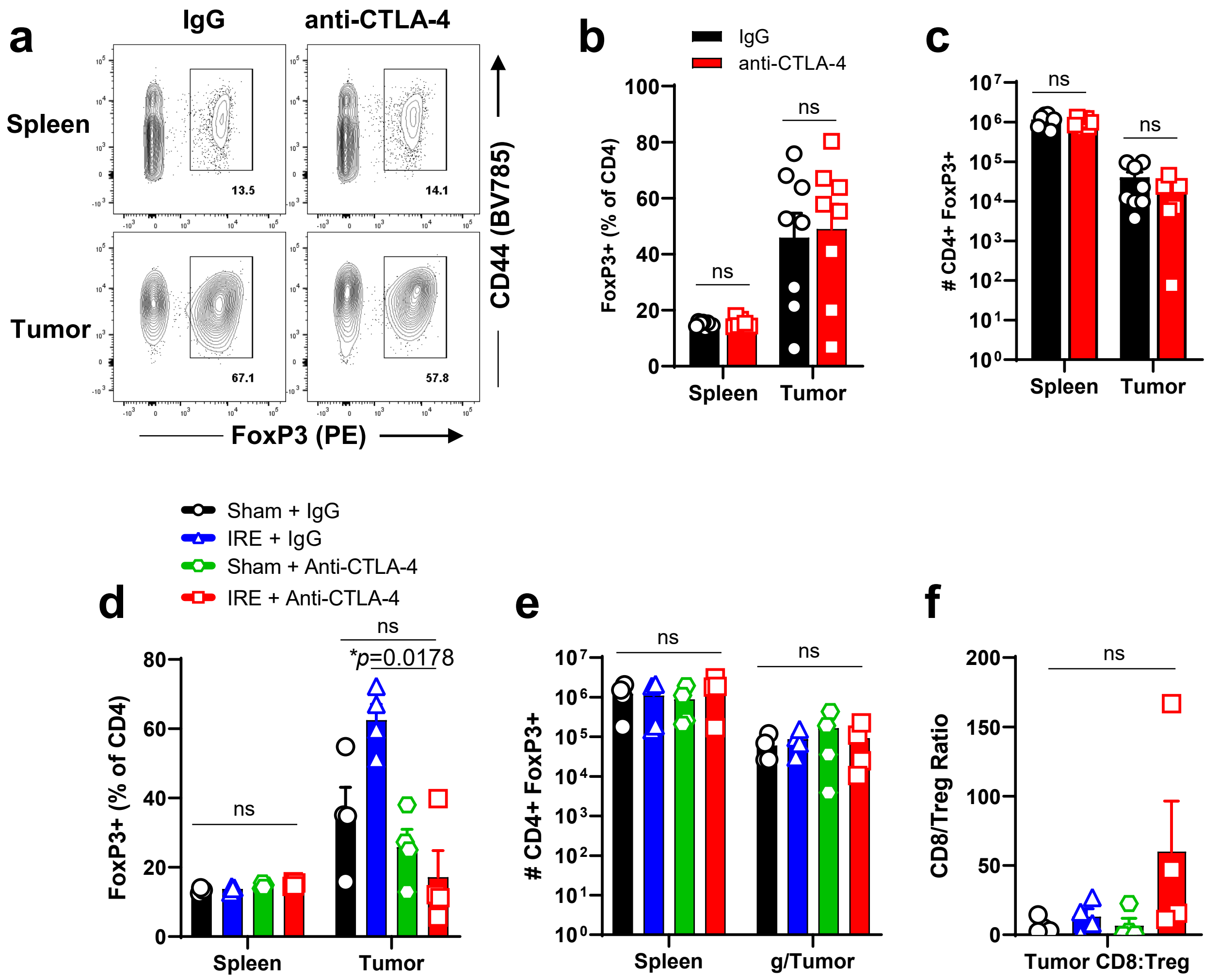

Supplementary Figure 6. Quantification of FoxP3+ regulatory T cells in TRAMP-C2 tumors following combination therapy. C57BL/6J mice were challenged s.c. with TRAMP-C2 tumor cells and mice bearing ~5mm diameter tumors were treated with Sham, IRE, and/or anti-CTLA-4 as described in Figure 6. Single cell suspensions from spleen and tumor were analyzed by flow cytometry on day 21 (**a-c**) or day 14 (**d-f**) following therapy onset. Thy1.2+ CD8- CD4+ T cells are shown. **a** Representative flow cytometry plots showing intracellular staining for FoxP3. **b, d** Frequency and **c, e** calculated numbers of FoxP3+ cells. **f** Ratio of total CD8+ T cells to total FoxP3+ T cells at day 14. Data points in **b-c** represent all individual mice (n=8/group) pooled from two independent experiments. Data points in **d-f** represent all individual mice (n=4/group) pooled from two independent experiments. Unpaired two-tailed T test with Holm-Sidak's correction was performed; *, $p<0.05$; ns, not significant. Bars represent mean ± S.E.M. Source data are provided as a Source Data File.

Supplementary Figure 7

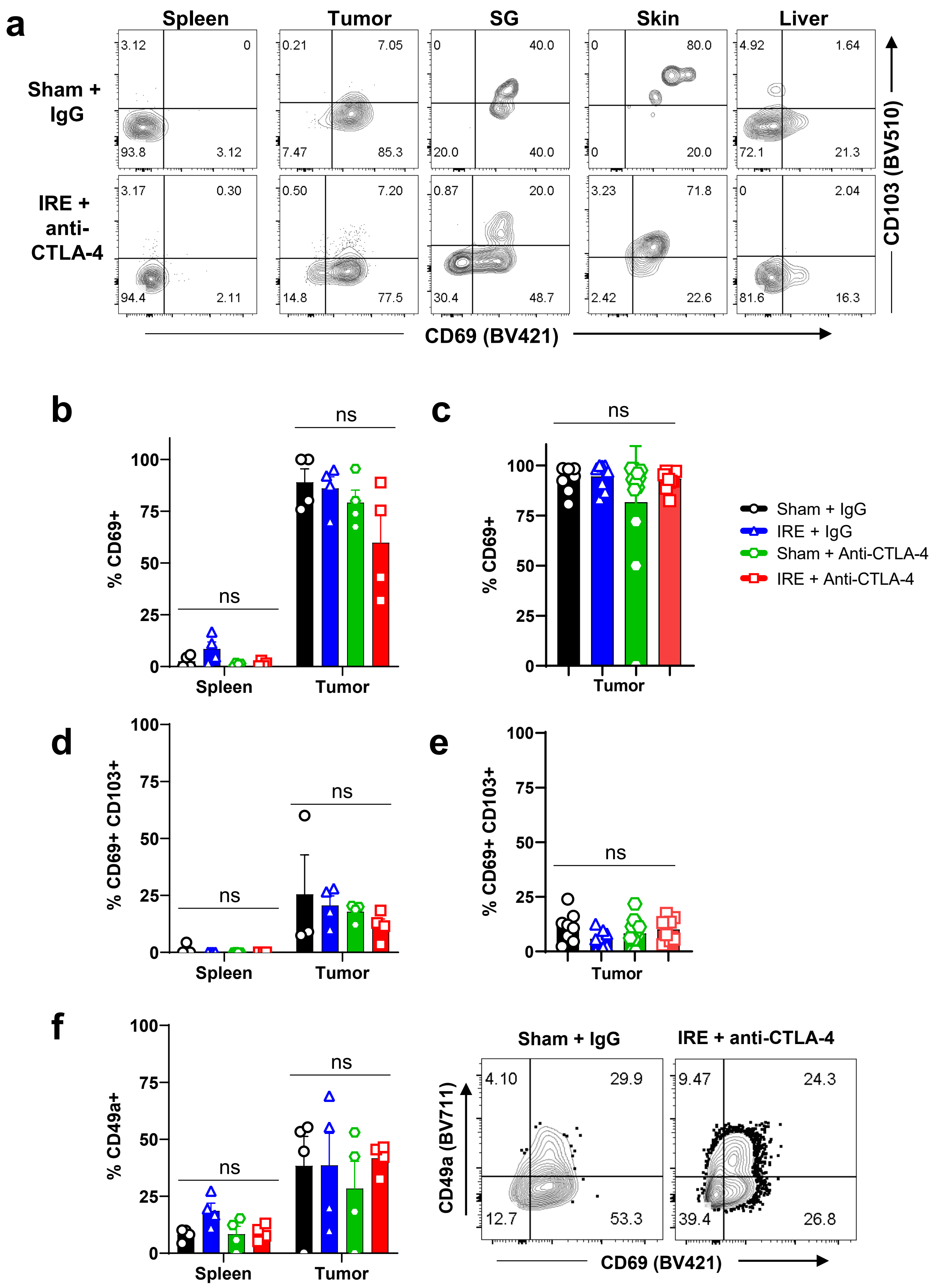

Supplementary Figure 7. Expression of tissue resident memory-associated molecules on SPAS-1+ T cells following combination therapy. C57BL/6J mice were challenged s.c. with TRAMP-C2 tumor cells and mice bearing ~5mm diameter tumors were treated with Sham, IRE, and/or anti-CTLA-4 as described in **Fig. 6**. **a** Representative flow cytometry plots depicting staining CD69 and CD103 at days 21 following therapy. Thy1.2+ CD8+ SPAS-1+ T cells from the IV-negative gate reflecting the tumor parenchyma are shown (see **Fig. 6d**). Single cell suspensions from the indicated tissues were analyzed by flow cytometry on day 14 (**b,d,f**) or day 21 (**a**,**c,e**) following therapy onset. **b-c** Frequency of CD69+ SPAS-1+ T cells in individual mice. **d-e** Frequency of CD103 expression at day 14 and 21, respectively. **f**, Frequency of CD49a expression at day 14, with representative gating showing in the right panels. Data points in **b,d,f** represent all individual mice (n=4/group)(n=3 for Sham + IgG tumor in **d)** pooled from two independent experiments performed at day 14. Data points in **c, e** represent all individual mice pooled from four independent experiments at day 21. Sham + IgG and IRE + IgG, n=8 mice/group; Sham + anti-CTLA-4, n=13 mice/group; IRE + anti-CTLA-4, n=11 mice/group for CD69 staining in **c** and n=8 mice/group shown for CD103 staining in **e**. Kruskal-Wallace test with Dunn's multiple comparison test was performed; ns, not significant. Bars represent mean ± S.E.M. SG, Salivary gland. Source data are provided as a Source Data File.

Supplementary Figure 8

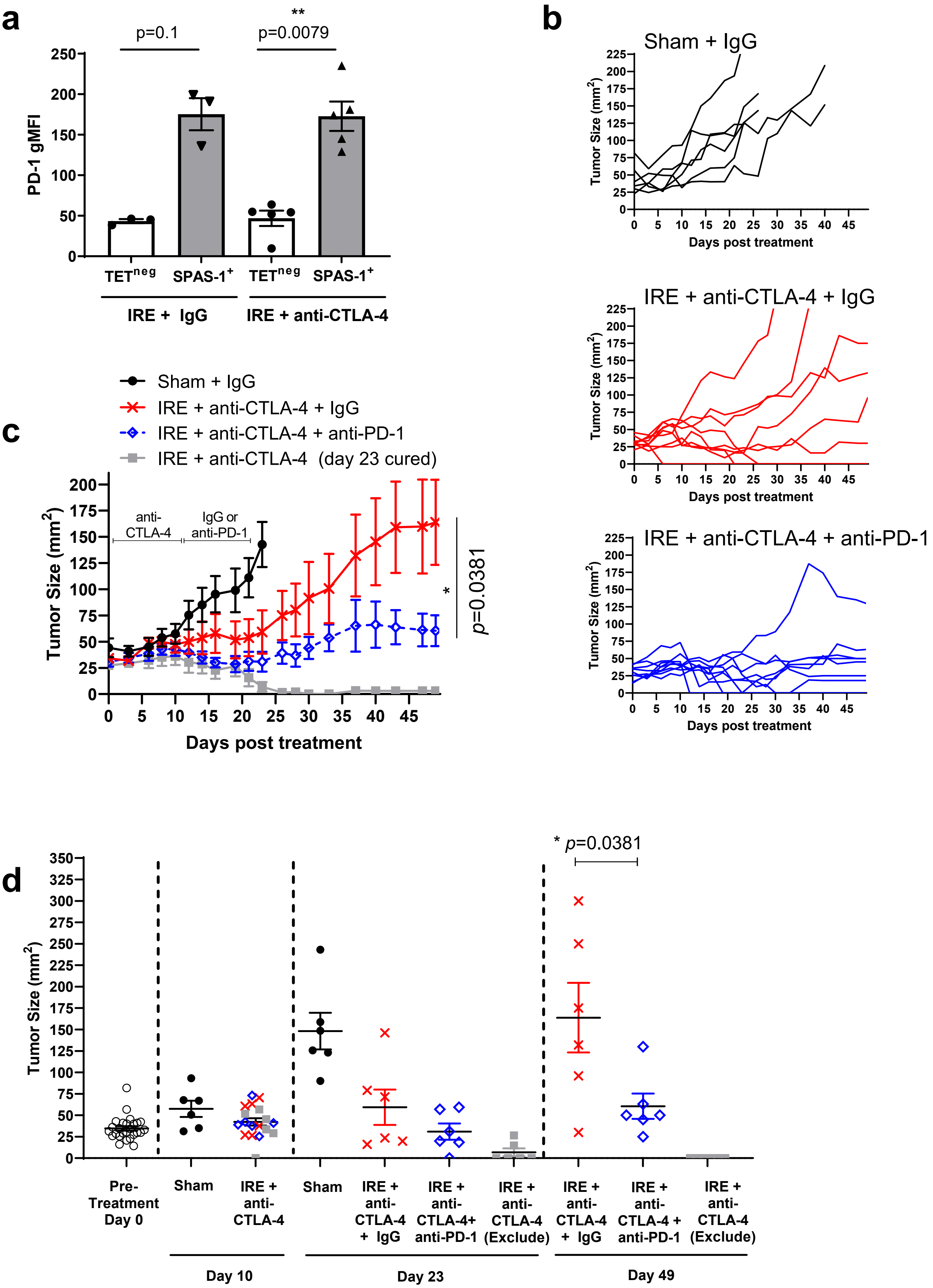

Supplementary Figure 8. Anti-PD-1 treatment following IRE plus anti-CTLA-4 combination therapy sustains tumor regression. **a** Flow cytometry of PD-1 expression on tetramer-negative and SPAS-1 tetramer+ CD8+ T cells. Samples reflect blood taken from individual mice at day 14 after the indicated treatment, from the experiment shown in **Fig. 5**, and are representative of similar analysis performed in two independent experimental cohorts. Data reflect n=3 mice/group for IRE + IgG, and n=5 mice/group for IRE + anti-CTLA-4 conditions. **b** Tumor growth curves for all individual mice shown in **Fig. 9d**. **c** Replot of all data shown in **Fig. 9d**, removing mice from both IRE + anti-CTLA-4 groups that cleared or nearly eliminated (< 9 mm^2) tumor by day 23 (shown retrospectively in gray). Data in **c** represent all individual mice (n=6/group) regrouped and pooled from the two independent cohorts in Fig. 9d. **d** Individual tumor sizes of mice replotted in **c**, showing days 0, 10, 23, and 49 following IRE treatment. Unpaired two-tailed Mann-Whitney test was performed in **a**. **c-d**, Unpaired two tailed student's T test. Bars in **d-e** represent mean ± S.E.M. *, $p<0.05$, **, $p<0.01$. Bars represent mean ± S.E.M. Source data are provided as a Source Data File.
