# Supplementary material for: Irreversible electroporation augments checkpoint immunotherapy in prostate cancer and promotes tumor antigen-specific tissue-resident memory CD8+ T cells
Source: Nat Commun. 2021 Jun 23;12:3862. doi: 10.1038/s41467-021-24132-6 (PMC8222297; doi:10.1038/s41467-021-24132-6)
Supplement: Supplementary file 2 — Reporting Summary [file 41467_2021_24132_MOESM2_ESM.pdf]

nature research

Corresponding author(s): Brandon J. Burbach

Last updated by author(s): May 16, 2021

Reporting Summary

Nature Research wishes to improve the reproducibility of the work that we publish. This form provides structure for consistency and transparency in reporting. For further information on Nature Research policies, see our Editorial Policies and the Editorial Policy Checklist.

Statistics

For all statistical analyses, confirm that the following items are present in the figure legend, table legend, main text, or Methods section.

n/a	Confirmed	
☐	☒	The exact sample size (n) for each experimental group/condition, given as a discrete number and unit of measurement
☐	☒	A statement on whether measurements were taken from distinct samples or whether the same sample was measured repeatedly
☐	☒	The statistical test(s) used AND whether they are one- or two-sided<br>*Only common tests should be described solely by name; describe more complex techniques in the Methods section.*
☐	☒	A description of all covariates tested
☐	☒	A description of any assumptions or corrections, such as tests of normality and adjustment for multiple comparisons
☐	☒	A full description of the statistical parameters including central tendency (e.g. means) or other basic estimates (e.g. regression coefficient) AND variation (e.g. standard deviation) or associated estimates of uncertainty (e.g. confidence intervals)
☐	☒	For null hypothesis testing, the test statistic (e.g. F, t, r) with confidence intervals, effect sizes, degrees of freedom and P value noted<br>*Give P values as exact values whenever suitable.*
☒	☐	For Bayesian analysis, information on the choice of priors and Markov chain Monte Carlo settings
☒	☐	For hierarchical and complex designs, identification of the appropriate level for tests and full reporting of outcomes
☒	☐	Estimates of effect sizes (e.g. Cohen's d, Pearson's r), indicating how they were calculated

Our web collection on statistics for biologists contains articles on many of the points above.

Software and code

Policy information about availability of computer code

Data collection	FACS Diva (version 8.0.1) for flow cytometry acquisition
Data analysis	Flow Jo (version 10) was used for flow cytometry visualization and analysis. GraphPad Prism (version 9) was used for graphing and statistical analysis.

For manuscripts utilizing custom algorithms or software that are central to the research but not yet described in published literature, software must be made available to editors and reviewers. We strongly encourage code deposition in a community repository (e.g. GitHub). See the Nature Research guidelines for submitting code & software for further information.

Data

Policy information about availability of data

All manuscripts must include a data availability statement. This statement should provide the following information, where applicable:

- Accession codes, unique identifiers, or web links for publicly available datasets
- A list of figures that have associated raw data
- A description of any restrictions on data availability

Source data are provided with this paper. Source data are available as a Source Data File. The remaining data that support the data are available within the Article, Supplementary Information or available from the authors upon reasonable request.

Field-specific reporting

Please select the one below that is the best fit for your research. If you are not sure, read the appropriate sections before making your selection.

- [x] Life sciences
- [ ] Behavioural & social sciences
- [ ] Ecological, evolutionary & environmental sciences

For a reference copy of the document with all sections, see nature.com/documents/nr-reporting-summary-flat.pdf

Life sciences study design

All studies must disclose on these points even when the disclosure is negative.

Sample size	Sample size was defined based on previous studies (eg Beura at al, Immunity, 2018; Rosato et al, Nature Communications, 2019). 4 to 8 mice per group was generally sufficient to detect differences between groups with power value 0.8 and 5% significant.
Data exclusions	No data were excluded
Replication	The number of independent experimental replicates is given in each figure legend, typically reflecting 1 to 3 repeat experiments. All reported attempts at replication were successful.
Randomization	Mice were enrolled for treatment when tumors reached a pre-designated size, and were equally distributed between each treatment group based on tumor size.
Blinding	Blinding was not possible because a single investigator coordinated technical aspects of all experiments in this study. Tumor measurements were taken without foreknowledge of groupings where possible, but visible features of animals in treatment cohorts made bona fide blinding unachievable due to limitations of staff technical coordination.

Reporting for specific materials, systems and methods

We require information from authors about some types of materials, experimental systems and methods used in many studies. Here, indicate whether each material, system or method listed is relevant to your study. If you are not sure if a list item applies to your research, read the appropriate section before selecting a response.

Materials & experimental systems

n/a	Involved in the study	
☐	☒	Antibodies
☐	☒	Eukaryotic cell lines
☒	☐	Palaeontology and archaeology
☐	☒	Animals and other organisms
☒	☐	Human research participants
☒	☐	Clinical data
☒	☐	Dual use research of concern

Methods

n/a	Involved in the study	
☒	☐	ChIP-seq
☐	☒	Flow cytometry
☒	☐	MRI-based neuroimaging

Antibodies

Antibodies used

CD8a FITC Clone 53-6.7 Tonbo Biosciences Cat# 35-0081-U100 Lot# C0081090518354
CD8a PE-Cy7 Clone 53-6.7 Tonbo Biosciences Cat# 60-0081-U100 Lot# C0081092917603
CD8a BV421 Clone 53-6.7 Biolegend Cat# 100753 Lot# B273942
CD8a BV785 Clone 53-6.7 Biolegend Cat# 100750 Lot# B273618
CD8a BUV737 Clone 53-6.7 BD Biosciences Cat# 564297 Lot# 9030634
CD4 BUV496 Clone GK1.5 BD Biosciences Cat# 612952 Lot# 0205396
CD44 FITC Clone IM7 Tonbo Biosciences Cat# 35-0441-U100 Lot# C0441121112354
CD44 BV785 Clone IM7 Biolegend Cat# 563736 Lot# B245972
CD45.1 BUV395 Clone A20 BD Biosciences Cat# 565212 Lot# 7258960
CD45.2 FITC Clone 104 Tonbo Cat# 35-0454-U100 Lot# C0454050614354
CD90.2 BUV395 Clone 53-2.1 BD Biosciences Cat# 565257 Lot# 8330989
CD90.2 AF700 Clone 30-H12 Biolegend Cat# 105320 Lot# B207170
CD62L BV605 Clone MEL-14 Biolegend Cat# 104438 Lot# B277998
CD69 BV421 Clone H1.2F3 Biolegend Cat# 104528 Lot# B208881
B220 APC-Cy7 Clone RA3-6B2 Tonbo Biosciences Cat# 25-0452-U100 Lot# C0452092018253
MHCII APC-Cy7 Clone M5/11 4.15.2 Tonbo Biosciences Cat# 25-5321-U100 Lot# C53211070418253
PD-1 CD279 PerCP-Cy5.5 Clone RMP1-30 Biolegend Cat# 109120 Lot# B308142
PD-1 CD279 PE-Cy7 Clone RMP1-30 Biolegend Cat# 109110 Lot# B263885

PD-1 CD279 BV785 Clone 29F.1A12 Biolegend Cat# 135225 Lot# B298447
TIM-3 CD366 BV421 Clone RMT3-23 Biolegend Cat# 119723 Lot# B302682
LAG-3 CD223 BV711 Clone C9B7W Biolegend Cat# 125243 Lot# B312124
CD103 BV510 Clone 2E7 Biolegend Cat# 121423 Lot# B285331
CD49a BV711 Clone Ha31/8 BD Biosciences Cat# 564863 Lot# 8269504
TOX ef660 Clone TXRX10 ThermoFisher Cat# 506502-82 Lot# 2183623
IFNg PerCP-Cy5.5 Clone XMG1.2 Biolegend Cat# 505822 Lot# B150656
TNFa BV421 Clone MPG-XT22 Biolegend Cat# 563387 Lot# 0016054
FoxP3 PerCP-Cy5.5 Clone 3G3 Tonbo Biosciences Cat# 65-5773 Lot#C5773021215652
FoxP3 PE Clone FJK-16s eBioscience Cat# 1205773-82 Lot# 4295309

Dilutions were 1:100 for all of the ABOVE fluorescent conjugated antibodies, except 1:50 for CD45.1-BUV395 and CD69-BV421 and 1:300 for Thy1.2 BUV-395 and AF700

anti-CD40 (Clone FKG4.5) Bio-X-Cell In Vivo MAb Cat # BE0016-2 Lot# 671717N1
anti-CTLA-4 (Clone 9H10) Bio-X-Cell In Vivo MAb Cat # BE0131 Lot# 608516M2B; #70401801; #660518J1; #608516M2B
anti-PD-1 (Clone RMP1-14) Bio-X-Cell In Vivo MAb Cat # BE0146 Lot# 717918D1; #717919M1; #640517M2
anti-PD-L1 (Clone 10F.9G2) Bio-X-Cell In Vivo MAb Cat # BE0101 Lot# 598816M1
Polyclonal Syrian Hamster IgG (Clone Syrian Hamster IgG) Bio-X-Cell In Vivo MAb Cat# BE0087 Lot# 1773116M2; #642017M1

Validation

All antibodies were validated by and purchased from the following commercial vendors as specified by the manufacturer. Antibodies from Biolegend, BD Biosciences, Tonbo Biosciences, eBioscience, ThermoFisher, and BioXCell were subject to quality control testing by the manufacturer and validation data including use on mouse cells is available on the vendor website for each product number listed. Antibodies were also validated in-house under the staining conditions used.

Eukaryotic cell lines

Policy information about cell lines

Cell line source(s)

TRAMP-C2 cells were obtained from the American Type Culture Collection (ATCC-CRL-2731)

Authentication

The cells were not authenticated

Mycoplasma contamination

Cells used within 4 passages of receipt from ATCC and were not tested for mycoplasma contamination

Commonly misidentified lines
(See ICLAC register)

No misidentified cell lines were used

Animals and other organisms

Policy information about studies involving animals; ARRIVE guidelines recommended for reporting animal research

Laboratory animals

C57BL/6J (CD45.2) and B6.SJL-PtprcaPepcb/BoyJ (CD45.1) mice (mus musculus) used in this study were from Jackson Laboratories. All mice were male except where indicated. Mice were 8-10 weeks of age at the time of study enrollment.

Wild animals

The study did not involve wild animals

Field-collected samples

The study did not involve animals collected from the field

Ethics oversight

Studies were approved by the Institutional Animal Care and Use Committee (IACUC) at the University of Minnesota

Note that full information on the approval of the study protocol must also be provided in the manuscript.

Flow Cytometry

Plots

Confirm that:

- [x] The axis labels state the marker and fluorochrome used (e.g. CD4-FITC).
- [x] The axis scales are clearly visible. Include numbers along axes only for bottom left plot of group (a 'group' is an analysis of identical markers).
- [x] All plots are contour plots with outliers or pseudocolor plots.
- [x] A numerical value for number of cells or percentage (with statistics) is provided.

Methodology

Sample preparation

Mice were euthanized by cervical dislocation under isoflurane anesthesia. In some experiments, 200 µl of PBS containing 3 micrograms of FITC or PE-Cy7 conjugated anti-CD8α antibody (clone 53-6.7, Tonbo Biosciences) was introduced into the bloodstream by retro-orbital injection 3 minutes prior to euthanasia, to mark circulating CD8+ T cells. Single cell suspensions were generated as previously described. Briefly, organs were collected in and all rinses performed using harvest buffer (HB)

consisting of RPMI-1640 (Corning) containing 5% FBS, 10mM HEPES and 4mM L-glutamine. Spleens and lymph nodes were dissociated by scraping over an etched plastic dish with a flat syringe plunger. Liver was pushed through a 70 µm strainer using a flat plunger, followed by isolation of lymphocytes from the interface of a 44%/66% Percoll step gradient. Blood, spleen, and liver samples were treated with hypotonic saline (ACK buffer) to lyse red blood cells. All other tissues were transferred into GentleMacs tubes (Miltenyi) containing 0.5mL of HB and rapidly minced into ~1 mm pieces using microdissection scissors. Digest Buffer (HB containing 1mM each of CaCl2 and MgCl2, and 1 µg/mL DNAse I (Sigma DN25) was supplemented with collagenase as follows and was added to each sample to 10 mL final volume, followed by incubation at 37°C in an angled rack with shaking at 250 rpm. Salivary glands and lungs were digested for 45mins and 60 mins, respectively, in 200U/mL of collagenase I (Worthington CLS-1). Kidney and female reproductive tract were digested for 30 mins and 60 mins, respectively, in 0.5 µg/mL of collagenase IV (Sigma C5138). Tumors were digested in 100U/mL of collagenase I and 0.5 mg/mL collagenase IV. Hair was shaved from a flank skin sample (distal from tumor, if present) and fat removed by scraping with a forcep handle. A 2 cm2 of skin was minced then added to 5 mL of digest buffer containing 2 mg/mL collagenase IV and 1 µg/mL DNAse for 60 mins at 37°C with shaking. Enzymatically digested tissues were further dissociated by running the Spleen.01 program on a GentleMacs dissociator (Miltenyi). All samples were then passed through 60 µm nylon mesh and resulting single cell suspensions were centrifuged and resuspended in a defined volume of FACS buffer (HBSS containing 0.1% sodium azide and 2% bovine serum), prior to being transferred to 96-well plates for staining with fluorescent antibody cocktails and MHC-I tetramers.

See also Methods section.

Instrument

Flow Cytometry was performed on BD LSRII and Fortessa flow cytometers equipped with 4 or 5 lasers, respectively

Software

FACS Diva software (Version 8.0.1) was used for acquisition and Flow Jo software (version 10) was used for analysis

Cell population abundance

The proportion of cells is displayed on flow cytometry plots and absolute counts were calculated using counting beads

Gating strategy

FSC-H/FSC-A gating was used to identify singlets of appropriate size for lymphocytes, followed by SSC-A/FSC-A to define a lymphocyte gate. All T cells were identified using positive staining for Thy1.2 (CD90.2) defined by baseline separation, and excluding cells staining positive for a dump channel (MHC-II, B220, and a viability dye to simultaneously exclude dead cells that may nonspecifically bind antibodies). Within the dump-negative, Thy1.2+ gate, CD4negative CD8+ T cells were identified by baseline separation. In many cases, within these CD8+ T cells, cells circulating in the vasculature were identified with an intravenous (IV) CD8+ T cell gate. MHC-I tetramers to SPAS-1 were used to identify the tumor-specific CD8+ T cells.

☒ Tick this box to confirm that a figure exemplifying the gating strategy is provided in the Supplementary Information.
